# Metabolomic Profiling Reveals Heat-Responsive Metabolic Markers in Honey Bee Workers Under Contrasting Diets

**DOI:** 10.3390/ijms27209237

**Published:** 2026-10-17

**Authors:** Hyeonji Jang, Hyunjee Kim, Olga Frunze, Hyung-Wook Kwon, Boyun Kim, Jewon Jung

**Affiliations:** 1 Department of SmartBio, College of Life and Health Science, Kyungsung University, Busan 48434, Republic of Korea; hyeonji00201@ks.ac.kr; 2 Department of Life Sciences and Convergence Research Center for Insect Vectors (CRCIV), Incheon National University R&D Complex, 265 Harmony-ro, Yeonsu-gu, Incheon 22014, Republic of Korea; beamed79@hanmail.net (H.K.); frunzeo1@inu.ac.kr (O.F.); hwkwon@inu.ac.kr (H.-W.K.); 3 College of Fisheries Science, Pukyong National University, 45 Yongso-ro, Nam-gu, Busan 48513, Republic of Korea

**Keywords:** *Apis mellifera*, acute heat stress, metabolomics, bee bread, sucrose, amino acid metabolism

## Abstract

Acute heat exposure challenges honey bee (*Apis mellifera*) workers, yet the associated small-molecule response and its relationship to nutritional status remain incompletely resolved. Workers were conditioned for 7 days on bee bread plus sucrose (B+S) or sucrose alone (S), maintained at 37 °C or exposed to 45 °C for 3 h, and thorax–abdomen metabolites were profiled by untargeted Ultra-performance liquid chromatography-quadrupole time-of-flight mass spectrometry (UPLC-QTOF/MS). Three independent cages were used per diet × temperature group, with 15 workers per cage pooled to generate one cage-level biological replicate (*n* = 3 per group). Putative metabolite annotation used accurate-mass (10 ppm) and isotope-pattern matching against METLIN and the Agilent Personal Compound Database and Library (PCDL), followed by manual curation, without tandem mass spectrometry (MS/MS) o or authentic-standard confirmation. After statistical filtering and biological curation, 149 heat-responsive metabolite annotations were retained under B+S and 123 under S. Targeted re-extraction of proteinogenic amino acids from the same raw files recovered 16 common peaks across all samples. Fifteen increased at 45 °C in both diets, whereas proline decreased; 14 of 16 showed a larger fold change under B+S. Phenylalanine-related metabolism showed the strongest convergence across targeted amino-acid analysis, untargeted phenylpyruvate detection, and Kyoto Encyclopedia of Genes and Genomes (KEGG) enrichment. Lipid-class responses also differed descriptively between diets, with a bias toward decreased free fatty acyls under B+S and decreased phospholipids under S. These findings define coordinated metabolic markers of acute heat exposure in worker bees and show that, within the targeted amino-acid dataset, response magnitudes were generally larger under B+S while retaining the same overall direction.

## 1. Introduction

Honey bees (*Apis mellifera*) are exposed to multiple environmental pressures that can converge within managed colonies. Climate warming and more frequent heat extremes increase the probability of acute thermal challenge, while periods of limited or nutritionally imbalanced forage can reduce the resources available to support stress responses [1,2,3,4,5,6]. Honey bee colonies buffer temperature through collective thermoregulation, including fanning, water collection, evaporative cooling, and endothermic heat production, but this buffering capacity is finite [7,8,9]. Consequently, nutritional condition and acute heat exposure represent two management-relevant stress axes that may act simultaneously on worker physiology.

The physiological consequences of heat stress in honey bees are increasingly well documented. Excessive heat can impair colony thermoregulation, reduce adult survival and colony population, constrain flight through metabolic heat production and water balance, compromise queen fertility, and reduce foraging motivation [10,11,12,13,14,15,16]. At the molecular level, the response is characterized by induction of heat-shock proteins, changes in immune signaling, and oxidative stress defenses [17,18,19,20,21]. These studies establish that high temperature affects honey bee performance from the molecular to the colony level, but they do not by themselves identify the small-molecule changes that could serve as practical indicators of acute thermal stress.

Nutritional state is likely to influence this metabolic response. Access to high-quality floral resources can reduce the effects of heat stress on bees [4], pollen protein and lipid content affects resilience to other stressors [5], and extreme heat can itself reduce pollen nutritional quality [3]. In addition, diet quality modifies metabolic and immune transcriptional responses of honey bees during acute heat exposure [6]. Thus, the metabolic consequences of heat should be considered in the context of the nutritional resources available before and during the thermal challenge.

Metabolomics provides a direct route to identifying such stress-responsive small-molecule signatures. Thermal stress studies in other insects have revealed coordinated changes in amino acids, energy substrates, and membrane-associated metabolites [22,23,24,25]. In honey bees, metabolomic analyses have described responses to pollen nutrition [26] and have shown that high-temperature/high-humidity shock alters sugars, polyols, and free amino acids [27]. However, studies explicitly aimed at identifying acute heat-responsive metabolic markers across contrasting nutritional conditions remain limited, particularly at the whole-body untargeted-metabolome level.

Accordingly, the present study aimed to identify metabolic markers associated with acute heat exposure in worker honey bees under two contrasting nutritional conditions. Workers were maintained from day 3 post-emergence on either bee bread plus sucrose (B+S) or sucrose alone (S), and then held at 37 °C or exposed to 45 °C for 3 h. Untargeted UPLC-QTOF/MS was used to define heat-responsive metabolite signatures within each dietary background. Because the untargeted datasets were processed as parallel within-diet temperature contrasts rather than as a formal diet-by-temperature interaction, nutritional effects on the magnitude of response were assessed more directly by re-extracting proteinogenic amino acids from all twelve raw files in a single targeted workflow. This design therefore prioritizes discovery of heat-responsive metabolic markers while using nutritional background as an important physiological context for interpreting the acute heat response.

## 2. Results

### 2.1. Acute Heat Exposure Produced Broad, Bidirectional Changes in the Worker Body Metabolome

**Figure 1:**
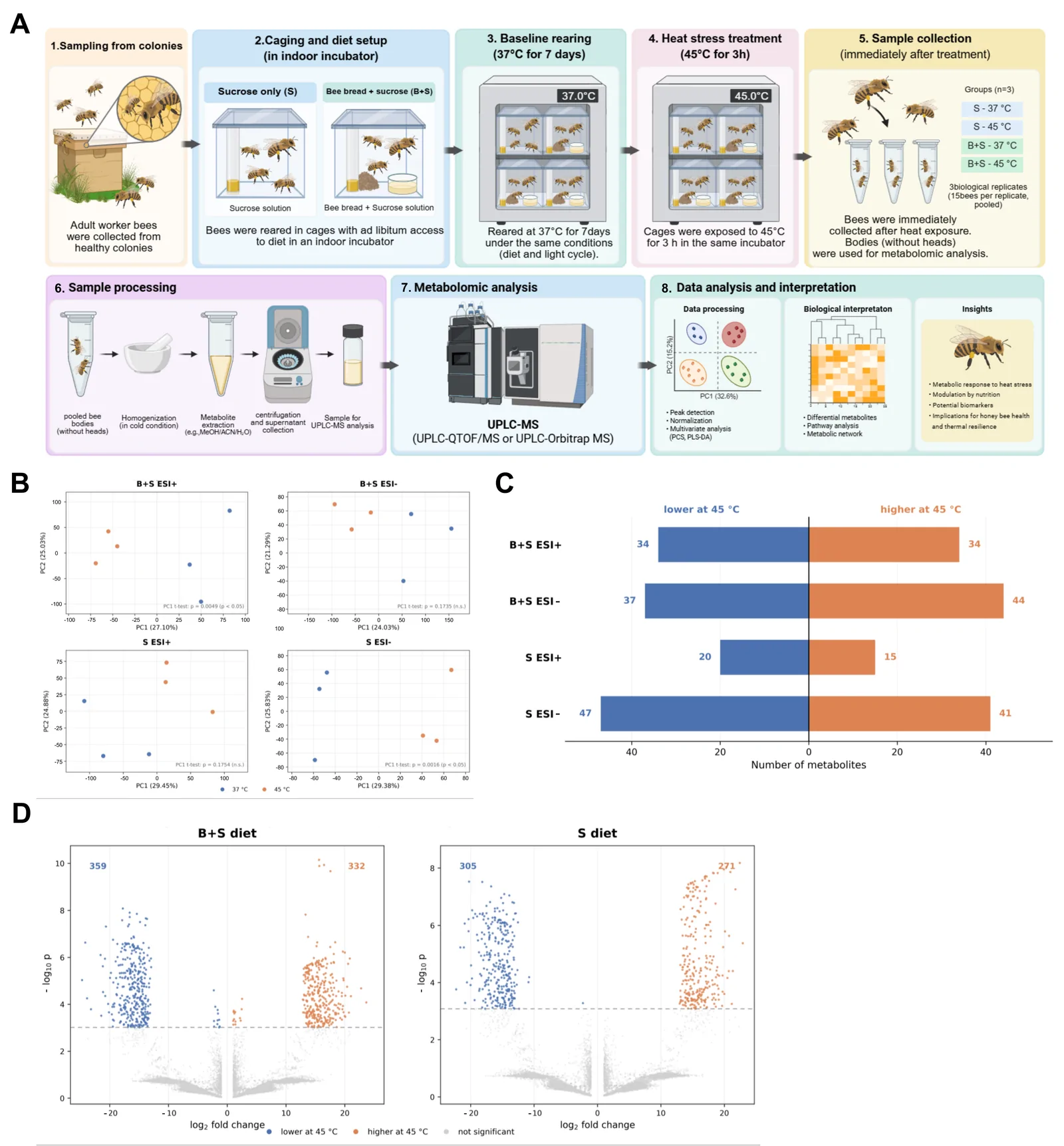
Experimental design and global metabolomic response of worker honey bees to acute heat exposure under two dietary backgrounds. (**A**) Newly emerged workers were conditioned for 7 d on either sucrose alone (S) or bee bread plus sucrose (B+S), then maintained at 37 °C or exposed to 45 °C for 3 h before immediate collection. Heads were removed and thorax–abdomen tissues were analyzed by untargeted UPLC-QTOF/MS in positive and negative electrospray ionization modes. Fifteen bodies collected from each independent cage were pooled for each biological replicate (*n* = 3 cages per diet–temperature combination). (**B**) Principal component analysis score plots for B+S ESI+, B+S ESI−, S ESI+, and S ESI− datasets. Blue and terracotta points denote 37 °C and 45 °C samples, respectively; axis labels report the variance explained by PC1 and PC2. PC1 scores were compared between temperatures using Welch’s *t*-test as a descriptive measure of separation (*n* = 3 biological replicates per temperature group); these *p* values were not used for feature selection. (**C**) Numbers of curated metabolites significantly lower or higher at 45 °C within each diet and ionization mode. Blue indicates lower abundance at 45 °C and terracotta indicates higher abundance at 45 °C. The four ion-mode counts sum to 149 curated significant metabolites for B+S and 123 for S. (**D**) Volcano plots generated after pooling the positive- and negative-mode entity tables within each diet and reanalyzing per-sample log_2_ abundances by Welch’s *t*-test with Benjamini–Hochberg correction. Blue and terracotta points denote entities lower and higher at 45 °C, respectively; gray points are not significant. Numbers at the upper left and right indicate the counts of significant entities in each direction. The dashed horizontal line marks the largest raw *p* value satisfying an FDR threshold of 0.05 within that diet. Entity counts in panel D exceed the curated named-metabolite counts in panel C because the volcano analysis retains the broader aligned entity set before biological curation. The exported entity tables had already been restricted to |log_2_ FC| ≥ 1 (FC ≥ 2 or FC ≤ 0.5) by the MPP workflow; the volcano plots therefore contain no entities inside that fold change window, which is visible as an empty vertical band on either side of zero.

The experimental workflow is summarized in Figure 1A. Workers were conditioned for 7 days on bee bread plus sucrose (B+S) or sucrose alone (S), after which diet-matched groups were maintained at 37 °C or exposed to 45 °C for 3 h and sampled immediately. Unsupervised PCA of the four ionization-mode datasets showed temperature-associated displacement of the biological replicates, with the clearest separation in the B+S ESI+ and S ESI− datasets (Figure 1B). The other two datasets showed weaker or partial separation, indicating that the magnitude of the multivariate temperature effect varied with ionization mode and dietary background rather than producing an identical global pattern in every dataset. After the MPP differential-abundance workflow and biological curation, 149 heat-responsive metabolite annotations were retained for B+S and 123 for S (Tables S1 and S2). Under B+S, 78 were higher and 71 were lower at 45 °C; under S, 56 were higher and 67 were lower. When resolved by ionization mode, B+S contained 34 lower and 34 higher metabolites in ESI+ and 37 lower and 44 higher metabolites in ESI−, whereas S contained 20 lower and 15 higher metabolites in ESI+ and 47 lower and 41 higher metabolites in ESI− (Figure 1C). Thus, neither diet showed a unidirectional loss or accumulation of metabolites after heat exposure. A separate fold change-filtered entity-level Welch-test/BH display, generated from the per-sample log_2_ abundance matrices and pooled across ESI+ and ESI− within each diet, showed the broader statistical signal before biological curation (Figure 1D). These entity tables had already been restricted to |log_2_ FC| ≥ 1 by the MPP workflow, so the display covers the fold change-filtered entity set rather than every detected feature. In this representation, 359 entities were lower and 332 were higher at 45 °C under B+S, while 305 were lower and 271 were higher under S. These volcano-plot counts describe the fold change-filtered entity-level statistical display and therefore are not expected to equal the curated metabolite counts in Figure 1C, Table 1 and Table 2, or Tables S1 and S2.

### 2.2. Amino Acids and Lipid-Related Classes Dominated the Curated Heat Response

**Figure 2:**
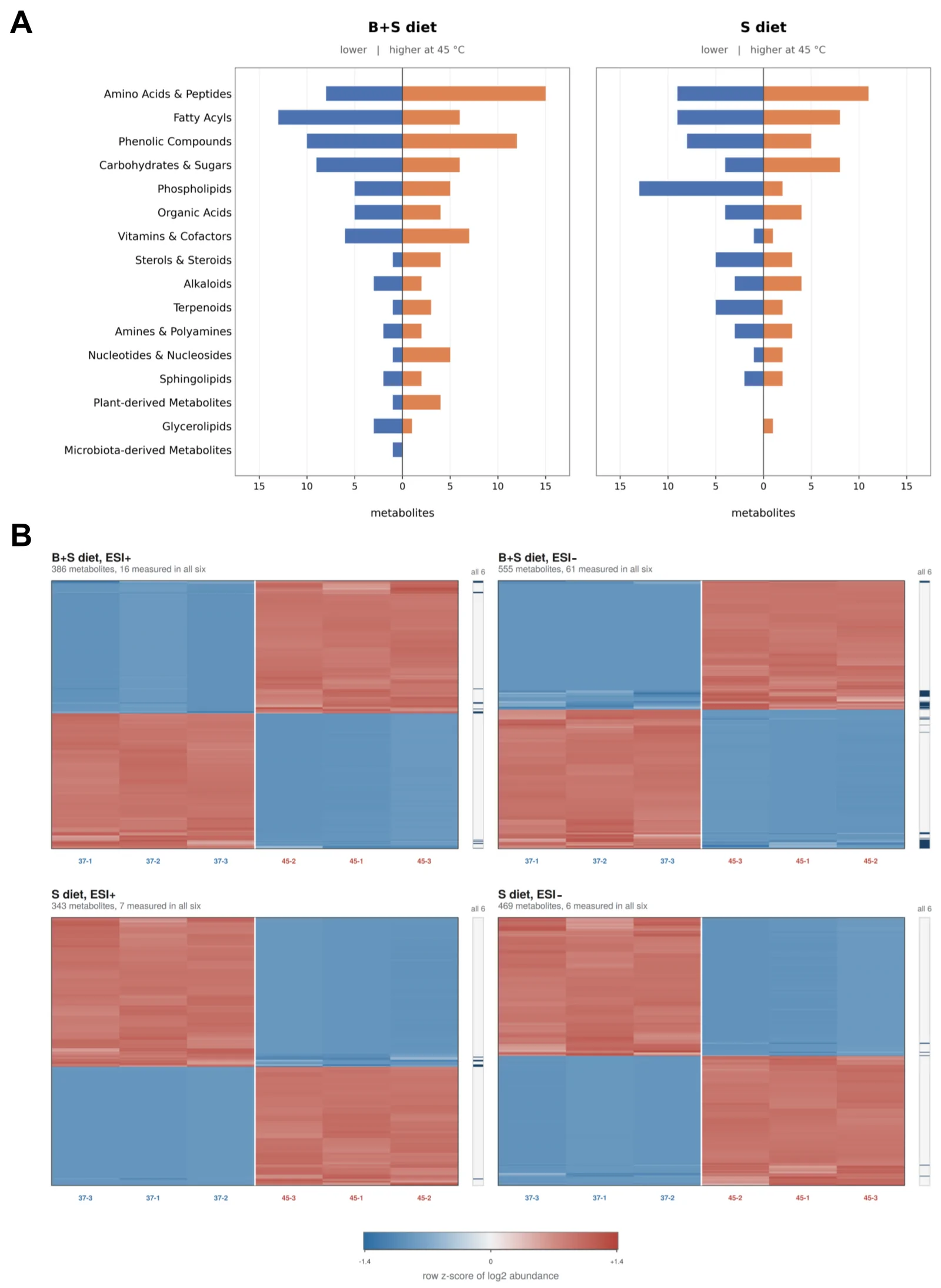
Chemical-class composition and hierarchical clustering of the heat-responsive metabolome. (**A**) Distribution of the curated significant metabolites among chemical super classes for B+S and S workers. Bars extending left (blue) indicate metabolites lower at 45 °C, whereas bars extending right (terracotta) indicate metabolites higher at 45 °C. The B+S list contained 149 metabolites (78 higher and 71 lower at 45 °C), and the S list contained 123 metabolites (56 higher and 67 lower). (**B**) Hierarchical heatmaps of the significant MPP entities in the four diet–ionization datasets (B+S ESI+, B+S ESI−, S ESI+, and S ESI−). These heatmaps show the complete significant MPP feature/entity set of each dataset (*n* = 386, 555, 343 and 469, respectively; 1753 in total) and are therefore larger than the curated annotation lists summarized in panel (**A**). Per-sample abundances were log_2_ transformed and standardized within each row; rows were ordered by hierarchical clustering using Pearson-correlation distance and average linkage. The color scale represents the row z-score of log_2_ abundance and was clipped at ±1.4 for display. Blue and red therefore indicate relatively lower and higher abundance within a given entity row, respectively, and should not be interpreted as absolute abundance across different entities.

The chemical-class composition of the curated responses is shown in Figure 2A. Amino acids and peptides constituted the largest responsive class in both diets, accounting for 23 B+S annotations (15 higher and 8 lower at 45 °C) and 20 S annotations (11 higher and 9 lower). Under B+S, other abundant responsive classes included phenolic compounds (22; 12 higher and 10 lower), fatty acyls (19; 6 higher and 13 lower), carbohydrates and sugars (15; 6 higher and 9 lower), and vitamins and cofactors (13; 7 higher and 6 lower). Under S, fatty acyls (17; 8 higher and 9 lower), phospholipids (15; 2 higher and 13 lower), phenolic compounds (13; 5 higher and 8 lower), and carbohydrates and sugars (12; 8 higher and 4 lower) were prominent. Two lipid-class patterns were particularly asymmetric. In the B+S group, 13 of 19 fatty acyl annotations decreased at 45 °C, whereas the ten B+S phospholipid annotations were evenly divided between increases and decreases. In contrast, 13 of 15 phospholipid annotations decreased under S. These class-level differences were descriptive because the two dietary datasets were processed independently and were not subjected to a formal diet-by-temperature interaction test. Hierarchical clustering of all significant MPP features/entities—386 for B+S ESI+, 555 for B+S ESI−, 343 for S ESI+ and 469 for S ESI−, 1,753 in total—produced temperature-associated abundance blocks in all four ionization-mode datasets (Figure 2B). Because the heat maps were generated from features already selected for differential abundance, the clustering is presented as a visualization of the internal structure of the selected response rather than as an independent significance test.

### 2.3. Metabolites Quantified in Every Replicate Identified Robust Heat-Responsive Candidates

**Figure 3:**
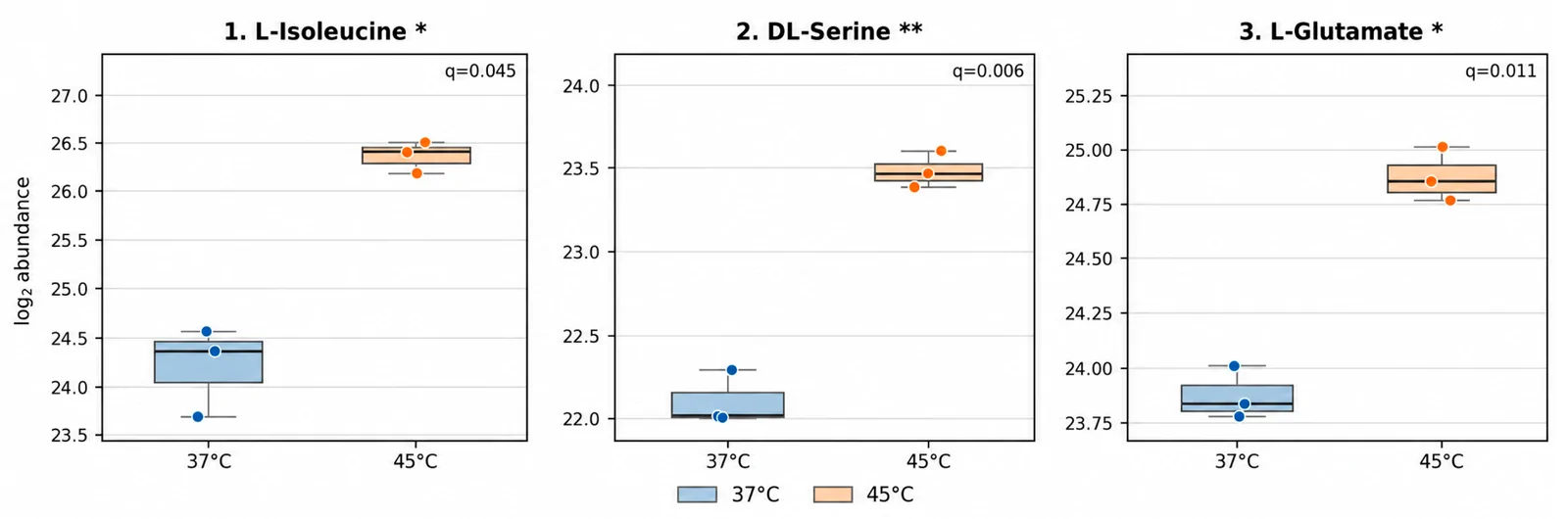
Representative heat-responsive metabolites quantified in every sample of both temperature groups. Box-and-point plots show log_2_ abundance at 37 °C (blue) and 45 °C (terracotta) for three biological replicates per temperature. The three displayed metabolites are L-isoleucine, DL-serine, and L-glutamate from the B+S dataset. Each panel displays the Benjamini–Hochberg-adjusted *p* value (*q*) from the two-group *t*-test implemented in MPP for the comparison of 45 °C versus 37 °C. Asterisks indicate *q* < 0.05 (*) or *q* < 0.01 (**). The remaining 12 representative metabolites are shown in Supplementary Figure S1.

To distinguish measured abundance gradients from presence–absence contrasts, we focused on metabolites detected in all three biological replicates at both temperatures. Twenty-one curated significant metabolites met this detection-frequency criterion, of which 15 were selected for detailed visualization to represent different chemical classes, directions of change, and dietary backgrounds. For clarity, Figure 3 presents the first three representative metabolites—L-isoleucine, DL-serine, and L-glutamate—all of which increased significantly at 45 °C under B+S (log_2_ FC = 2.15, 1.38, and 1.00, respectively; BH-adjusted *p* < 0.05). The remaining 12 representative metabolites are shown in Supplementary Figure S1. These include additional B+S metabolites, such as L-phenylalanine, phenylpyruvic acid, D-sorbitol, and several other heat-responsive compounds, as well as the two S-diet representatives, PI(18:3/0:0) and nitiducarpin (Figure S1; Table 1 and Table 2). The full curated metabolite lists, including features expressed primarily as presence–absence contrasts, are provided in Tables S1 and S2.

### 2.4. KEGG Enrichment Converged on Aromatic Amino-Acid Metabolism Under B+S

**Figure 4:**
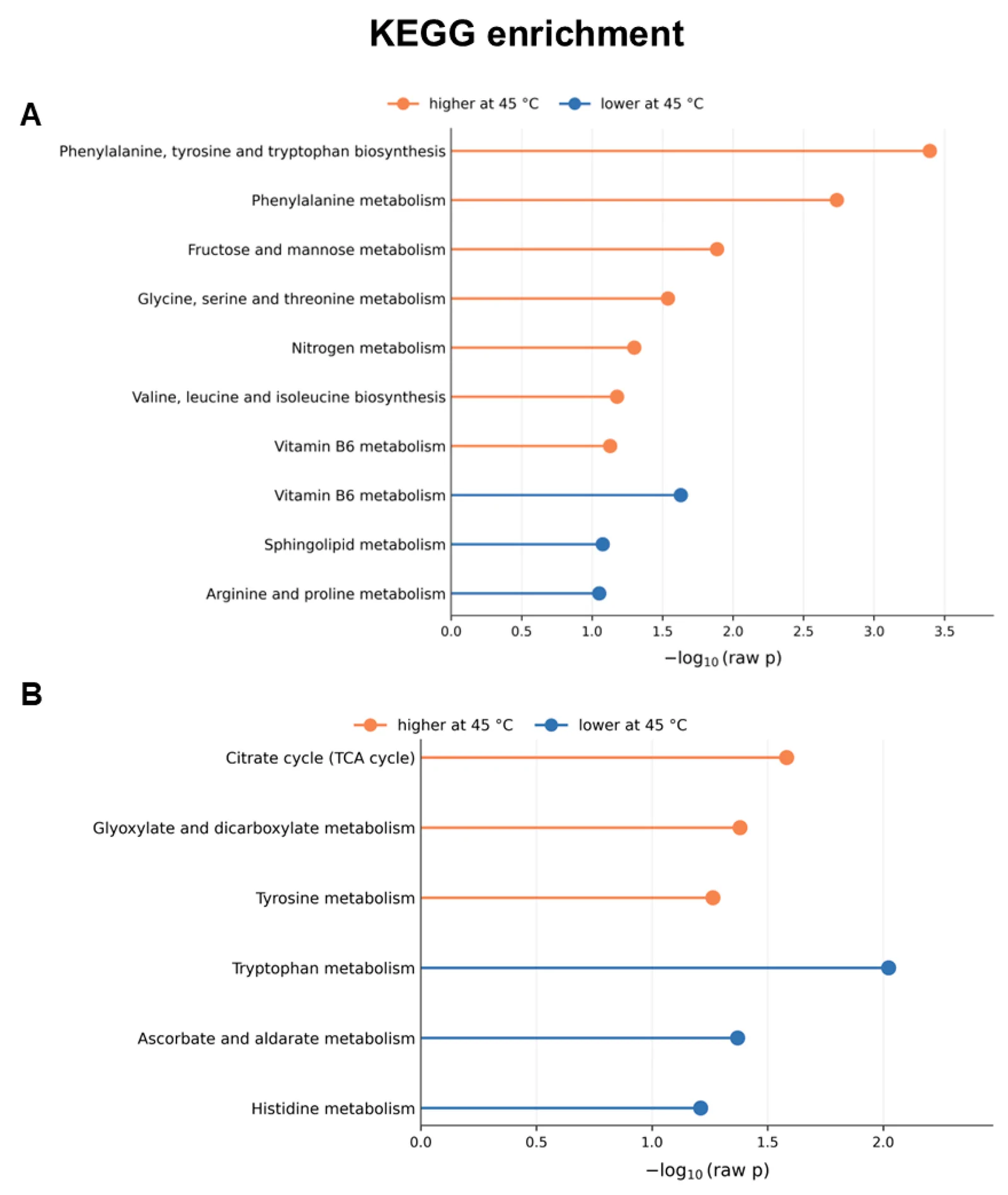
KEGG pathway over-representation analysis of metabolites altered by acute heat exposure. Curated metabolites carrying KEGG identifiers were analyzed separately after division into higher-at-45 °C and lower-at-45 °C lists for each diet. (**A**) B+S diet. (**B**) S diet. The x-axis shows −log10 (raw *p*) from the MetaboAnalyst over-representation analysis; terracotta denotes pathways represented among metabolites higher at 45 °C and blue denotes pathways represented among metabolites lower at 45 °C. The plots are intended as an exploratory summary of pathway-level tendencies and therefore display raw *p* values without an on-figure significance threshold. Multiple-testing correction was evaluated separately; phenylalanine, tyrosine and tryptophan biosynthesis in the B+S higher-at-45 °C list was the only pathway that remained significant after Benjamini–Hochberg correction (FDR = 0.0326).

KEGG over-representation analysis was performed separately for metabolites higher and lower at 45 °C within each diet (Figure 4). For visualization, pathways with raw *p* < 0.10 were ranked by −log10 (raw *p*); multiple-testing correction was retained for inferential interpretation. Among B+S metabolites that increased at 45 °C, phenylalanine, tyrosine and tryptophan biosynthesis was the strongest pathway (raw *p* = 0.000402; FDR = 0.0326; 2 hits in a 4-compound pathway) and was the only pathway in the four analyses that remained significant after BH correction. Phenylalanine metabolism was the next-ranked B+S increase (raw *p* = 0.00184; FDR = 0.0745), followed by fructose and mannose metabolism, glycine/serine/threonine metabolism, nitrogen metabolism, branched-chain amino-acid biosynthesis, and vitamin B6 metabolism (Figure 4A).

For B+S metabolites that decreased, vitamin B6 metabolism, sphingolipid metabolism, and arginine and proline metabolism were the highest-ranked pathways, but none survived multiple-testing correction. Under S, citrate-cycle and glyoxylate/dicarboxylate metabolism were the leading pathways among metabolites that increased, while tryptophan metabolism was the strongest pathway among metabolites that decreased (raw *p* = 0.00949); again, none of the S-diet pathways met FDR < 0.05 (Figure 4B). The limited number of mapped compounds in several pathways, often one or two hits, constrains pathway-level inference.

### 2.5. Targeted Re-Extraction Revealed a Coordinated Free-Amino-Acid Response with Generally Larger Fold Changes Under B+S

**Figure 5:**
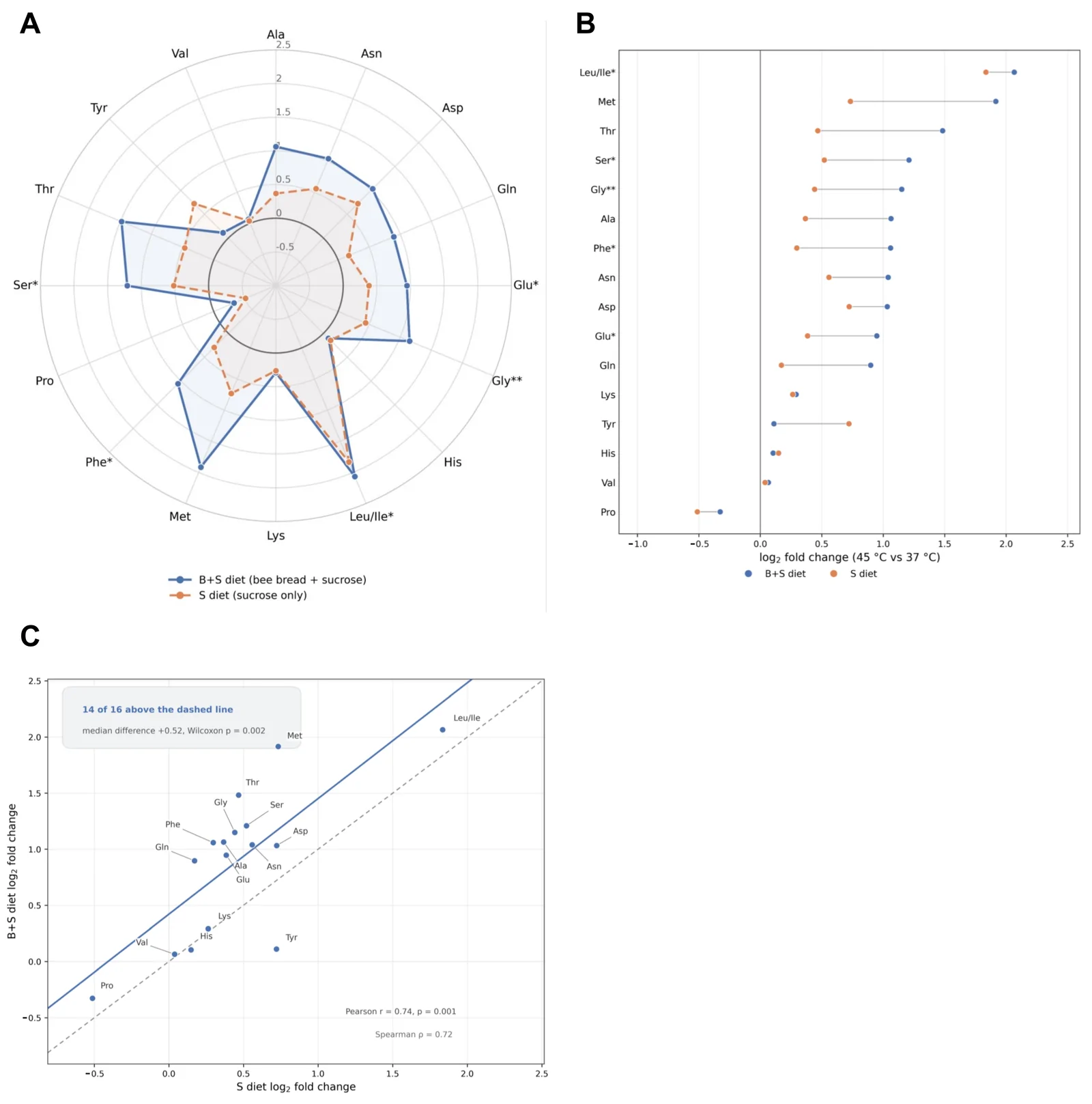
Targeted re-extraction of free amino acids reveals a coordinated heat response with generally larger response amplitudes under B+S. Sixteen proteinogenic amino-acid peaks were recovered in all three replicates of both temperature groups under both diets; leucine and isoleucine were isobaric and are reported together as Leu/Ile. (**A**) Radar plot of log_2_ fold change (45 °C versus 37 °C) for the sixteen amino acids under B+S (blue) and S (terracotta). Fifteen of sixteen amino acids increased at 45 °C in each diet, whereas proline decreased in both. (**B**) The same log_2_ fold changes ranked by the B+S response. Each horizontal line links the B+S and S values for the same amino acid. Asterisks appended to amino-acid labels denote Benjamini–Hochberg significance for the B+S comparison (* *q* < 0.05; ** *q* < 0.01); no amino acid reached *q* < 0.05 under S. (**C**) Direct comparison of diet-specific response amplitudes. Each point represents one amino acid, with the S-diet log_2_ fold change on the x-axis and the B+S log2 fold change on the y-axis. The dashed line denotes equal response in the two diets (y = x), and the solid line is the least-squares fit. Fourteen of sixteen amino acids lay above the line of equality; the median B+S-minus-S difference was 0.52 log_2_ units (paired Wilcoxon signed-rank *p* = 0.002). The diet-specific fold changes were correlated (Pearson r = 0.74, *p* = 0.001; Spearman ρ = 0.72).

Targeted extraction recovered 16 of the 20 proteinogenic amino acids as common peaks measured in every replicate of both temperature groups and both dietary backgrounds; leucine and isoleucine were not chromatographically resolved and are reported together as Leu/Ile. The response was highly directional: 15 of 16 amino acids were higher at 45 °C in each diet, and proline was the only amino acid to decrease in both (exact sign test *p* = 0.000519; Figure 5A,B). Under B+S, Leu/Ile showed the largest increase (log_2_ FC = 2.07), followed by methionine (1.92) and threonine (1.48), whereas proline decreased by 0.33 log_2_ units. Under S, Leu/Ile also showed the largest increase (1.83), while proline decreased by 0.51 log_2_ units.

Five amino acids reached BH-adjusted *p* < 0.05 under B+S: glycine (*q* = 0.0079), Leu/Ile (*q* = 0.0205), serine (*q* = 0.0205), glutamate (*q* = 0.0205), and phenylalanine (*q* = 0.0424). None reached adjusted significance under S, with glutamate the closest (*q* = 0.0779). Despite the difference in individual significance, the response ranks were concordant between diets (Pearson *r* = 0.739, *p* = 0.00107; Spearman *ρ* = 0.718, *p* = 0.00175; Figure 5C).

**Figure 6:**
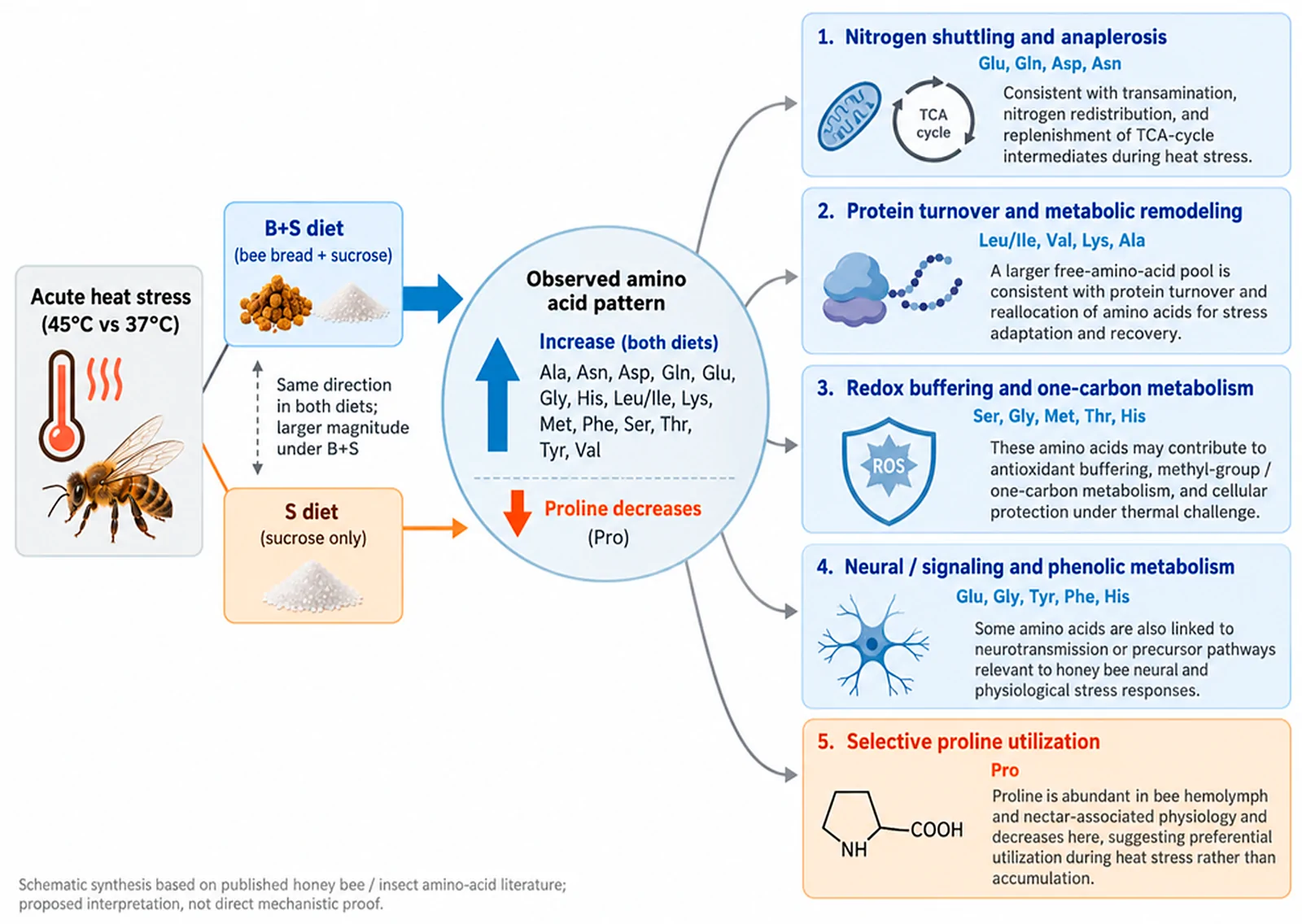
Literature-informed working model for the biological interpretation of the amino-acid response. The schematic groups observed increase in the free amino-acid pool into putative functions related to nitrogen shuttling and anaplerosis, protein turnover and metabolic remodeling, redox buffering and one-carbon metabolism, and neural/signaling or phenolic metabolism, while highlighting the selective decrease in proline. This figure is interpretive and does not represent direct measurements of flux, proteolysis, redox state, neural activity, or pathway activity in the present experiment.

Heat-associated amino-acid fold changes were generally larger under B+S than under S. Fourteen of the 16 amino acids lay above the line of equal response in the B+S-versus-S comparison, and the median paired difference was +0.522 log_2_ units (paired Wilcoxon signed-rank *p* = 0.00214). Thus, within this targeted common feature space, the B+S condition was associated with larger heat-response amplitudes while the overall response direction remained conserved. Figure 6 presents a literature-informed, hypothesis-generating model for the biological interpretation of this coordinated amino-acid response and should not be regarded as direct mechanistic evidence.

## 3. Discussion

### 3.1. Acute Heat Causes Broad Metabolic Redistribution and Lipid Remodeling

Three hours at 45 °C altered a large and chemically diverse set of curated metabolite annotations under both dietary backgrounds: 149 under B+S and 123 under S. The direction of change was approximately balanced under B+S (78 higher, 71 lower) and shifted modestly toward decreases under S (56 higher, 67 lower). This bidirectional structure argues against interpreting the response as a simple global depletion and instead indicates redistribution among metabolic pools. The broader entity-level volcano plots likewise contained substantial numbers of both increases and decreases, although those counts arise from a different statistical representation and should not be conflated with the curated lists. This pattern is consistent with metabolomic studies of thermal stress in other insects, in which heat produces coordinated changes in energy metabolism and amino-acid pools rather than uniform loss [22,23,24,25]. Cross-species comparisons of effect magnitude should be made cautiously because temperature, duration, tissue composition, and analytical workflows differ, but the present data demonstrate that the adult honey bee worker body is metabolically labile over a 3 h thermal episode. The 21 frequency-complete significant metabolites provide the most directly quantitative subset of the untargeted data; the 15 representatives shown in Figure 3 include multiple amino acids or immediate derivatives and foreshadow the targeted amino-acid result.

The clearest class-level asymmetries involved lipids. Under B+S, 13 of 19 fatty acyl annotations decreased at 45 °C, whereas phospholipids were evenly split between increases and decreases. Under S, the strongest directional bias occurred in glycerophospholipids, with 13 of 15 decreasing. Because the untargeted B+S and S datasets were processed independently, these patterns should be interpreted as within-diet responses rather than as formal interaction. Nevertheless, they nominate different lipid pools as major components of the heat response under the two nutritional backgrounds. Depletion of free fatty acyls under B+S is compatible with increased lipid utilization, conversion into complex lipids, or stress-related lipid signaling. Thermal stress is known to draw down energy reserves in insects [25,28], and free fatty acids participate in both energy supply and signaling [29,30]. The present experiment did not measure β-oxidation or flux, so the decrease should not be assigned to a single fate. Under S, the coordinated loss of phosphatidylethanolamine-, phosphatidylinositol-, and phosphatidylserine-related features is consistent with acute membrane remodeling and/or membrane damage. Homeoviscous adaptation provides a framework for regulated lipid remodeling with temperature [31,32,33,34,35], while oxidative stress provides a parallel mechanism through lipid peroxidation [20,21]. The 3 h design cannot distinguish these processes.

Several individual lipid annotations reinforce the need for caution. Dehydrophytosphingosine moved in opposite directions in the two curated datasets, while glucosylsphingosine decreased under B+S. Sphingolipid signaling is involved in insect heat responses [36], but a single putative metabolite cannot define pathway direction. Likewise, decreases in a cardiolipin species and an ether-linked lysophosphatidylethanolamine under B+S are potentially relevant to mitochondrial and membrane stability [37,38,39], particularly because thorax-containing samples are rich in flight muscle, yet these assignments remain MSI level 2–3. Prostanoid- and oxylipin-like annotations were therefore retained as candidate stress-related lipid signals without assigning mammalian stereochemistry or mechanism [40,41].

### 3.2. Free Amino Acids and Aromatic Amino-Acid Metabolism Define the Dominant Heat-Response Axis

Amino acids and related compounds formed the largest responsive chemical class in both diets. The targeted re-extraction was important because most free amino acids had been removed by upstream frequency filtering in the untargeted workflow. Re-interrogating all twelve raw files in one common feature space recovered 16 amino-acid peaks in every replicate of both diets and temperatures. Fifteen increased at 45 °C in each diet, while proline decreased in both. Five amino acids met BH-adjusted significance under B+S (glycine, Leu/Ile, serine, glutamate, and phenylalanine), and four of these were also represented independently among the frequency-complete untargeted metabolites. This convergence across distinct extraction/processing routes makes expansion of the free-amino-acid pool the best-supported biochemical axis in the study.

A larger free-amino-acid pool after acute heat can arise from several non-exclusive processes, including increased protein turnover, transient suppression of protein synthesis, altered transport or redistribution of amino acids between tissues and hemolymph, redistribution of nitrogen, and changes in amino-acid oxidation. Such changes may also reflect metabolic reorganization during acute thermal stress, because amino acids can serve as substrates for gluconeogenic and other energy-related pathways in insects [22,23,24,25,42]. The present experiment measures pool size rather than flux and therefore cannot determine the relative contribution of these mechanisms. Additional contributions from heat-induced protein damage followed by proteolysis, membrane damage or cellular leakage, and matrix-dependent ion suppression or enhancement during LC–MS analysis also cannot be excluded. Nevertheless, the pattern is consistent with the previous observation that high-temperature shock remodels free amino acids together with sugars and polyols in A. mellifera [27]. It also differs from Aedes aegypti, where heat depletes glutamate and glutamine and promotes branched-chain amino-acid degradation [22], emphasizing that the sign of amino-acid responses depends on species, tissue, nutritional state, and exposure regime. Proline was the sole directional exception and deserves separate consideration because it is widely recognized as a stress-associated compatible osmolyte in insects. However, proline responses are not uniformly accumulative and can vary according to the type and duration of stress. Previous insect studies have shown that proline may accumulate during acclimation to some environmental stresses but can also be utilized during subsequent heat hardening [43]. In honey bees, hemolymph proline decreases after exhaustive flight, consistent with some utilization during high energetic demand [44]. However, direct respirometry of permeabilized A. mellifera flight muscle found little capacity for proline-supported respiration compared with bumblebees and wasps [45]. Thus, the decrease observed here does not necessarily conflict with a stress-protective role for proline, but may reflect stress-dependent utilization, redistribution among tissues or hemolymph, protein incorporation, osmotic functions, or other metabolic demands. The present data therefore identify proline as a selectively depleted pool rather than demonstrating its specific metabolic fate. Resolving this mechanism will require isotope tracing or time-resolved targeted analysis.

The broader untargeted dataset shows that amino-acid-related metabolism was not uniformly expanded. Under S, kynurenine and melatonin decreased, while L-DOPA, prephenate, and several tyrosine-related annotations increased. The tryptophan–kynurenine pathway has established roles in insect pigmentation, redox biology, and microbiota–host signaling [26,46,47,48,49,50,51,52,53,54,55,56,57], and melatonin can contribute to thermal resilience in Apis cerana [58,59,60]. These annotations therefore support a stress-sensitive aromatic-amino-acid axis, but they do not demonstrate flux through a specific branch. Similarly, the concurrent decrease in histamine-related annotations and increase in N-acetyl-L-histidine under S is suggestive of altered histidine handling, yet 1-methylhistamine is an unusual insect annotation and should be considered provisional [61,62,63].

Phenylalanine provides the strongest point of convergence. Free phenylalanine increased in the targeted analysis under both diets and reached adjusted significance under B+S; phenylpyruvate increased in the untargeted B+S dataset; and aromatic amino-acid biosynthesis was the only KEGG enrichment surviving multiple-testing correction. This agreement does not prove activation of de novo phenylalanine biosynthesis, because pool expansion can also reflect protein turnover or altered catabolism, but it identifies this branch as a priority for targeted validation. The proposed physiological roles of the broader amino-acid response are summarized schematically in Figure 6; that panel is a hypothesis-generating framework rather than an additional experimental result.

Over-representation analysis produced one statistically supported pathway: phenylalanine, tyrosine and tryptophan biosynthesis among B+S metabolites that increased at 45 °C (raw *p* = 0.000402, FDR = 0.0326). Phenylalanine metabolism ranked second (raw *p* = 0.00184, FDR = 0.0745). These results are concordant with the free-phenylalanine and phenylpyruvate observations and therefore function best as corroboration of an independently visible metabolite signal.

All other displayed pathways should be treated as exploratory. The curated KEGG lists were small, and only a fraction of the responsive annotations mapped into the reference pathway library. Most displayed pathways were supported by one or two matched compounds, and none of the S-diet pathways survived multiple-testing correction. Consequently, lack of enrichment cannot be interpreted as lack of biological involvement, and pathway-level hypotheses should be validated with targeted metabolite panels or flux measurements rather than inferred from the over-representation output alone.

### 3.3. Energy, Redox, Endocrine, and Host–Microbiome Signatures of Acute Heat

Several organic acids and carbohydrates changed strongly, although many of the largest fold changes reflected features detected predominantly in one temperature group. Under S, aconitic acid increased while 2-methyleneglutarate decreased; under B+S, maleic acid increased and 3-ethylmalate decreased. These observations are compatible with altered central-carbon metabolism but cannot be mapped directly onto TCA-cycle flux from pool sizes alone. The TCA-cycle enrichment seen among S metabolites that increased at 45 °C is therefore best regarded as a candidate energy metabolism signal rather than direct evidence of increased cycle throughput. The most secure carbohydrate observations were among frequency-complete B+S metabolites: D-sorbitol increased and cellobiose decreased. The sorbitol response agrees with previous honey bee heat-shock data showing increases in sugars and polyols [27], consistent with their roles in osmotic protection and carbohydrate homeostasis. B+S also showed concurrent increases in pyridoxine, lipoamide, and dexpanthenol-related annotations. These cofactors are relevant to transamination, redox chemistry, and CoA-dependent metabolism [64,65,66], respectively; their co-occurrence with the enlarged free-amino-acid pool is mechanistically plausible but remains an association because neither enzyme activity nor cofactor flux was measured.

Several observations are compatible with oxidative stress during the 3 h exposure. Under B+S, an 8-hydroxyguanosine annotation increased together with changes in membrane lipids, while under S the antioxidant-related metabolite melatonin decreased. Oxidized guanine species are established markers of nucleic-acid oxidation [67], and heat-induced ROS formation is well documented in insects [20,21]. However, the present experiment did not measure ROS, antioxidant enzymes, lipid peroxidation, or authentic-standard-confirmed oxidative markers. These compounds therefore nominate an oxidative-load axis rather than demonstrate it.

The S dataset contained a strongly increased feature annotated as (10S)-juvenile hormone III diol, while B+S contained a more modest increase in 2-deoxyecdysone 22-phosphate. Acute stress can change juvenile hormone titres in workers [68,69], and adult honey bees retain active ecdysteroid signaling [70,71,72]. Nevertheless, the putative JH-diol annotation is not straightforward because JH degradation in *A. mellifera* is dominated by the esterase pathway and juvenile hormone epoxide hydrolase appears to have limited contribution [73,74]. These endocrine-related features should therefore be treated as candidates for authentic-standard confirmation rather than as evidence for a defined hormonal mechanism.

Thorax–abdomen metabolomics inevitably integrates host, dietary, and microbial metabolites. Several retained annotations, including an acyl-homoserine-lactone-like feature, a UDP-MurNAc pentapeptide, and acetoin, have plausible microbial origins [75,76,77,78,79,80]. The honey bee gut microbiota makes defined contributions to host carbohydrate and amino-acid metabolism [81,82], and temperature can alter symbiont functional output [83]. We did not profile microbial composition or perform germ-free controls, so these compounds cannot be assigned to the microbiota experimentally. Their value in the present dataset is as candidate markers of a host–microbiome response axis that can be tested directly in future work.

### 3.4. Targeted Free-Amino-Acid Responses Were Generally Larger Under B+S than Under S

The untargeted design does not support a quantitative metabolite-by-metabolite comparison between B+S and S because the two dietary backgrounds were processed as separate feature-alignment experiments. Class composition can be compared descriptively, but absence of the same annotation from the two curated lists cannot be interpreted as diet specificity. This distinction is essential when considering apparent opposite responses or limited overlap between the untargeted lists. The targeted amino-acid extraction removes this limitation for one pre-specified chemical class. The same 16 peaks were quantified in all twelve samples, allowing direct paired comparison of fold changes. The two diets produced the same directional pattern (15 increases and one decrease), and response ranks were strongly correlated, but 14 of 16 responses were larger under B+S and the median difference was +0.52 log_2_ units. Within this targeted dataset, heat-associated fold changes were generally larger under B+S while maintaining the same directional pattern. A bee-bread-containing diet supplies protein and free amino acids as well as lipids, sterols, micronutrients, and phytochemicals, so the mechanism cannot be attributed to protein alone. The larger excursion could reflect a larger mobilizable amino-acid/protein reserve, different rates of turnover or utilization, or broader differences in metabolic state. The conclusion should not be generalized to the rest of the metabolome without reprocessing all four groups in a single feature space and fitting an explicit diet-by-temperature interaction.

### 3.5. Limitations and Implications

Several limitations define the scope of these conclusions. First, the untargeted B+S and S datasets were processed independently; only the targeted amino-acid workflow provides a common feature space for quantitative diet comparison. Second, Figure 3 is restricted to metabolites quantified in every sample, whereas many large fold changes in Tables S1 and S2 are presence–absence-dominated contrasts. Third, all metabolite identities are putative MSI level 2–3 annotations based on accurate mass, isotope pattern, and database matching without authentic-standard confirmation [84,85]; this limitation also applies to the targeted amino-acid extraction, and Leu/Ile were not chromatographically resolved. Fourth, electrospray adducts, fragments, dimers, and duplicate annotations mean that feature counts can exceed the number of unique metabolites [86]. Fifth, pathway inference is constrained by limited KEGG coverage and small hit counts. Sixth, the experiment did not include survival, feeding, behavior, ROS, or recovery measurements, so the metabolic state cannot be placed precisely relative to the lethal threshold or separated into damage, acclimation, and recovery components. Finally, three biological replicates per group, each composed of 15 pooled workers from one independent cage, limit statistical power and do not estimate between-colony variation [87]. Because the present analysis excluded the head, complementary head-specific metabolomics would also be valuable for resolving heat- and diet-associated changes in neural, sensory, and neuroendocrine metabolism. These constraints make targeted validation across additional colonies, temperatures, and time points the necessary next step.

### 3.6. Summary of Key Discussion Points

#### 3.6.1. Metabolic and Lipid Remodeling

• Finding: Acute heat exposure induced broad, bidirectional metabolic changes under both dietary conditions.
• Evidence: A total of 149 and 123 curated heat-responsive metabolite annotations were identified under B+S and S, respectively, with decreased free fatty acyls predominating under B+S and decreased phospholipids under S.
• Interpretation: These patterns are consistent with metabolic redistribution and lipid remodeling rather than simple global metabolite depletion.
• Caution: The lipid-class differences between diets are descriptive because the untargeted datasets were processed independently and were not tested using a formal diet-by-temperature interaction.

#### 3.6.2. Free Amino Acids and Aromatic Amino-Acid Metabolism

• Finding: Expansion of the free-amino-acid pool was the most consistent metabolic response to acute heat, with proline as the major directional exception.
• Evidence: Fifteen of 16 commonly detected amino acids increased at 45 °C under both diets, and phenylalanine-related metabolism showed convergence across targeted phenylalanine, untargeted phenylpyruvate, and KEGG enrichment.
• Interpretation: The pattern is compatible with heat-induced changes in protein turnover, nitrogen redistribution, amino-acid utilization, and related metabolic remodeling.
• Caution: Pool-size measurements do not establish metabolic flux or mechanism, and alternative processes such as altered transport, cellular damage, or analytical effects cannot be excluded.

#### 3.6.3. Energy, Redox, Endocrine, and Host–Microbiome Responses

• Finding: Acute heat was accompanied by coordinated changes in metabolites associated with central-carbon metabolism, oxidative stress, endocrine signaling, and possible microbial metabolism.
• Evidence: Changes included organic acids and carbohydrates, oxidative-stress-related annotations, hormone-related features, and metabolites with plausible microbial origins.
• Interpretation: These observations nominate several additional physiological axes that may contribute to the acute heat response.
• Caution: These signals remain hypothesis-generating because flux, ROS, hormone levels, microbial composition, and authentic-standard confirmation were not directly assessed.

#### 3.6.4. Nutritional Modulation of the Amino-Acid Response

• Finding: The direction of the heat-induced amino-acid response was highly conserved between diets, but its magnitude was generally larger under B+S.
• Evidence: Fourteen of 16 amino acids showed larger heat-associated fold changes under B+S, with a median B+S-minus-S difference of +0.52 log_2_ units.
• Interpretation: Bee-bread supplementation may modify the metabolic capacity or reserve available during acute heat stress without fundamentally changing the direction of the amino-acid response.
• Caution: This effect cannot be attributed specifically to dietary protein, and it should not be generalized to the whole metabolome without a unified factorial analysis across all four experimental groups.

## 4. Materials and Methods

### 4.1. Honey Bees, Dietary Treatments and Acute Thermal Exposure

Worker bees were raised from brood produced by disease-free, queen-right colonies maintained at the Incheon National University experimental apiary (Incheon, Republic of Korea). Bee handling and cage-rearing procedures were adapted from OECD Test Guideline No. 213 [88] and the caged-worker procedure used by the same group [19]. To synchronize worker age, a frame of capped brood close to emergence was moved indoors and maintained overnight at 34.5 ± 0.5 °C and 75 ± 5% relative humidity (RH). Workers emerging during the subsequent 24 h were retained under the same conditions until day 3 post-emergence.

On day 3, workers were randomly allocated to plastic insect breeding cages (120 × 80 mm; SPL-TDS-ISBDJ, Pocheon, Republic of Korea) and assigned to one of two diets. The B+S diet supplied bee bread and 30% (*w*/*v*) sucrose solution simultaneously from separate feeders, both ad libitum. The S diet supplied 30% (*w*/*v*) sucrose solution alone. Thus, the dietary contrast represents the complete nutrient contribution of bee bread (including protein, free amino acids, lipids, sterols, vitamins, minerals, and phytochemicals) superimposed on sucrose, rather than an isolated protein-versus-carbohydrate comparison. Diets were renewed every 2 days, and cages were held in darkness at 37 ± 1 °C and 75 ± 5% RH for 7 days.

On day 7 of dietary treatment (day 10 post-emergence), each dietary group was divided between two temperature treatments. Control cages remained at 37 ± 1 °C, whereas heat-exposed cages were transferred to 45 ± 1 °C for 3 h. Humidity, darkness, and access to the assigned diet were maintained during exposure. The experiment therefore comprised two diets and two temperatures with three independent cages per diet–temperature combination, with each cage serving as one biological replicate. Because the untargeted B+S and S datasets were processed independently downstream, the temperature effect was analyzed within each diet rather than by a formal two-factor interaction. Immediately after exposure, 15 surviving workers were collected from each cage, immersed in liquid nitrogen, and stored at −80 °C. Caging, dietary treatment, thermal exposure, and sampling were performed at Incheon National University; frozen samples were transported on dry ice to Kyungsung University for extraction and mass-spectrometric analysis.

### 4.2. Metabolite Extraction

Metabolite extraction was restricted to the body compartment. Heads were removed from frozen workers by micro-dissection before homogenization, leaving thorax and abdomen for analysis. Heads were removed to focus the analysis on the thorax–abdomen compartment, which contains major tissues involved in energy metabolism and nutrient processing, including flight muscle, fat body, and the digestive tract. Because neural and sensory tissues in the head have distinct metabolic characteristics, their inclusion in a whole-body extract could dilute or obscure compartment-specific metabolic responses. The 15 bodies collected from each cage were pooled to form one biological replicate, yielding three independent cage-level replicates per diet-by-temperature combination and twelve samples in total. Each pool received 1 mL methanol–chloroform–water (3:1:1, *v*/*v*/*v*) and was homogenized for 3 min using a Beadbug microtube homogenizer (Benchmark Scientific Inc., Sayreville, NJ, USA). After 20 min at room temperature, samples were centrifuged at 20,000× *g* for 5 min at 4 °C. Supernatants were passed through 0.22 μm polyvinylidene fluoride membranes (Ultrafree-MC, Millipore, Burlington, MA, USA) and analyzed without further delay.

### 4.3. Untargeted LC-QTOF/MS Metabolomics

Analyses were run on an Agilent 1290 Infinity UHPLC coupled to an Agilent 6545 quadrupole time-of-flight mass spectrometer (Agilent Technologies, Santa Clara, CA, USA) at the Metabolomics Research Center for Functional Materials, Kyungsung University, with an electrospray ionization source operated in positive (ESI+) and negative (ESI−) mode in separate acquisitions. Positive electrospray ionization preferentially detects metabolites that readily protonate or form cationic adducts, including many amino acids, amines, and selected lipid species, whereas negative electrospray ionization favors acidic and readily deprotonated metabolites, such as organic acids, fatty acids, phosphorylated metabolites, and several phospholipid and phenolic species. The two ionization modes were therefore used complementarily to broaden metabolome coverage. Separation was carried out on an InfinityLab Poroshell 120 HILIC-Z column (2.1 × 100 mm, 2.7 μm; Agilent Technologies) held at 25 °C for positive-mode and at 50 °C for negative-mode runs, with an injection volume of 3 μL and a constant flow of 0.25 mL/min. Each biological replicate was analyzed using three technical LC–MS injections in each ionization mode, and the resulting peak-abundance values were averaged to generate a single abundance value per biological replicate before downstream statistical analysis.

For positive mode, solvent A was 10 mM ammonium formate in water with 0.1% formic acid and solvent B was 10 mM ammonium formate in 90% acetonitrile with 0.1% formic acid. The gradient started at 2% A and was held to 3 min, raised to 30% A at 11 min, 40% A at 12 min and 95% A at 16 min, held at 95% A to 18 min, returned to 2% A at 19 min and held to 20 min, with re-equilibration at 2% A completed by 24 min. For negative mode, solvent A was 10 mM ammonium acetate in water adjusted to pH 9.0 and solvent B was 10 mM ammonium acetate in 85% acetonitrile at pH 9.0. That gradient started at 4% A held to 2 min, rose to 12% A at 5.5 min and was held to 8.5 min, stepped to 14% A at 9 min and held to 14 min, then ramped to 18% A at 17 min and 35% A at 23 min, held to 24 min, returned to 4% A at 24.5 min and re-equilibrated by 29 min. Capillary voltage was 3.0 kV in positive and 3.5 kV in negative mode, and spectra were collected from *m/z* 50 to 1600.

### 4.4. Feature Processing, Annotation and Differential Abundance

All twelve samples were acquired within one analytical batch, and ion intensities were scaled to total ion current before downstream processing. Features were extracted in MassHunter Qualitative Analysis (v10.0, Agilent Technologies, Santa Clara, CA, USA) and putatively annotated by accurate-mass and isotope-pattern matching against METLIN and the Agilent Personal Compound Database and Library using a mass tolerance of 10 ppm. Annotated feature tables were transferred to Mass Profiler Professional (MPP v14.0, Agilent Technologies, Santa Clara, CA, USA) for filtering, scaling, alignment, and within-diet statistical testing. The untargeted workflow was based on full-scan MS1 data; MS/MS spectral matching and authentic-standard confirmation were not performed.

Because the two dietary backgrounds were processed as separate analytical experiments, four datasets were carried forward (B+S ESI+, B+S ESI−, S ESI+, and S ESI−), each analyzed as an independent 37 °C versus 45 °C contrast. Within each dataset, the 37 °C and 45 °C groups were compared using the two-group *t*-test implemented in MPP, followed by Benjamini–Hochberg (BH) correction. Features were retained when the temperature fold change satisfied FC ≥ 2 or FC ≤ 0.5 (|log_2_ FC| ≥ 1) and the BH-adjusted *p* value was <0.05. Compound identities were assigned in MPP ID Browser. Positive- and negative-ion tables were combined within each diet, after which unannotated *m/z* features, formula-only entries, short peptides/dipeptides, and database matches without a plausible endogenous, dietary, or gut-microbial origin were removed unless supported by additional evidence. Remaining annotations were assigned to chemical super classes according to LIPID MAPS, HMDB, and KEGG conventions. This curation yielded 149 annotations under B+S and 123 under S (272 in total), which form the basis of Table 1, Table 2, Tables S1 and S2, and the curated counts in Figure 1C and Figure 2A. Because one metabolite can produce multiple LC-MS features through adducts, in-source fragments, or dimers, these values are best interpreted as curated metabolite annotations rather than guaranteed counts of unique chemical species.

For the PCA, volcano plots, and hierarchical clustering displays, per-sample log_2_ abundances were exported from MPP and analyzed from the corresponding source matrices. The PC1 scores shown in Figure 1B were compared between 37 °C and 45 °C by Welch’s *t*-test as a descriptive summary of separation; these *p* values were not used for metabolite selection. The pooled volcano representation in Figure 1D used Welch’s *t*-test for each entity and BH correction across ESI+ and ESI− within a diet; it is a fold change-filtered entity-level statistical display and is distinct from the MPP-curated metabolite list used for Table 1 and Table 2 and Figure 1C and Figure 2A. The entity tables exported from MPP for this display had already been restricted by the MPP workflow to |log_2_ FC| ≥ 1 (FC ≥ 2 or FC ≤ 0.5), so they are not the complete unfiltered feature list; the Welch/BH statistics were recomputed on this fold change-pre-filtered entity set, and the plots consequently contain no entities inside that fold change window. Hierarchical clustering used Pearson-correlation distance with average linkage on row-wise *z*-scores; the selected heat-map features had already passed differential-abundance filtering, so the clustering was used descriptively rather than as an independent test of treatment separation.

### 4.5. Targeted Re-Extraction of the Free Amino Acids

Because several free amino acids were not retained in the aligned untargeted feature tables used for differential testing, this class was re-interrogated directly from the raw acquisition files. Batch Targeted Feature Extraction in MassHunter Profinder (v10.0, Agilent Technologies, Santa Clara, CA, USA) was run across all twelve .d files against the nineteen molecular formulas that cover the twenty proteinogenic amino acids; leucine and isoleucine are isobaric, share one formula and were not chromatographically resolved; they are reported together as Leu/Ile. Extraction used a mass tolerance of 10 ppm with no retention-time restriction, and considered the [M+H]^+^, [M+Na]^+^ and [M+K]^+^ adducts in positive mode and the [M−H]^−^, [M+HCOO]^−^ and [M+CH_3_COO]^−^ adducts in negative mode.

Profinder returns each formula as several retention-time groups. Groups lying within 0.20 min and 25 ppm of one another were merged, giving 247 merged groups. An amino acid was carried forward only when the same peak was recovered in both dietary backgrounds at retention times agreeing within 0.25 min and was measured in all three replicates of both temperature groups; where more than one peak met these conditions, the one with the largest total integrated area was taken. Sixteen of the twenty amino acids satisfied the criteria. Candidate chromatographic peaks and m/z assignments were manually inspected across samples, and retention-time consistency was considered during peak selection. Peak areas were log_2_-transformed, compared between temperature groups within each diet by Welch’s *t*-test, and corrected by the Benjamini–Hochberg procedure across the sixteen amino acids within a diet. Agreement between the two diets was assessed on the sixteen paired log_2_ fold changes by Pearson and Spearman correlation, by a paired Wilcoxon signed-rank test, and by an exact sign test on the number of amino acids rising at 45 °C. Because this extraction was based on accurate mass and molecular formula and was not confirmed by MS/MS or authentic standards, the amino-acid assignments, including phenylalanine and proline, are reported as putative annotations.

### 4.6. Pathway Enrichment Analysis

Pathway-level analysis used curated metabolites with a KEGG compound identifier, separated into four lists by diet and direction of change: B+S higher at 45 °C (46 identifiers), B+S lower (28), S higher (28), and S lower (32). Where the same KEGG identifier was associated with features moving in both directions within a diet, the direction represented by the better-supported feature was retained, prioritizing detection across more samples and then the smaller adjusted *p* value.

Each list was uploaded to MetaboAnalyst 6.0 as KEGG identifiers and analyzed separately by over-representation analysis against the KEGG pathway library. BH-adjusted *p* values were retained for inference. For Figure 4, pathways with raw *p* < 0.10 were displayed in ranked order to show the structure of the enrichment output, but no pathway was considered statistically supported unless it also passed BH correction. Coverage was evaluated because it limits pathway-level sensitivity: 39–59% of curated metabolites in the four lists carried a KEGG identifier, and only a subset of these identifiers mapped to the pathway library. Consequently, pathway enrichment was treated as corroborative rather than as a stand-alone discovery analysis.

### 4.7. Statistical Analysis

The independent cage was considered the experimental unit, with three biological replicates per diet-by-temperature combination (*n* = 3). Each biological replicate consisted of a pooled thorax–abdomen sample from 15 workers. For untargeted metabolomics, the four datasets (B+S ESI+, B+S ESI−, S ESI+, and S ESI−) were analyzed independently as 37 °C versus 45 °C comparisons. Differential features were evaluated using the two-group *t*-test implemented in Mass Profiler Professional, followed by Benjamini–Hochberg (BH) correction. Features were retained when |log_2_ fold change| ≥ 1 and the BH-adjusted p value was <0.05. For PCA, PC1 scores were compared between temperatures using Welch’s *t*-test as a descriptive measure of group separation; these *p* values were not used for metabolite selection. Entity-level volcano plots were generated using Welch’s *t*-test followed by BH correction of log_2_-transformed abundance values. Targeted amino-acid peak areas were log_2_-transformed and compared between temperatures within each diet using Welch’s *t*-test followed by BH correction across the 16 amino acids. Concordance of heat-response magnitudes between diets was evaluated using Pearson and Spearman correlations, and differences in fold change magnitude between diets were assessed using a paired Wilcoxon signed-rank test. An exact sign test was used to evaluate the directional predominance of amino-acid increases at 45 °C. Pathway over-representation analysis was performed in MetaboAnalyst 6.0, with BH-adjusted *p* < 0.05 considered statistically significant. All statistical tests were two-sided unless otherwise stated.

## 5. Conclusions

Acute exposure to 45 °C for 3 h produced broad, chemically structured changes in the body metabolome of adult honey bee workers under both bee-bread-supplemented and sucrose-only diets. The most reproducible response was an expansion of the free-amino-acid pool: 15 of 16 commonly recovered amino acids increased under both diets, proline decreased in both, and heat-associated fold changes were generally larger under B+S than under S. Untargeted metabolomics independently identified heat-responsive amino-acid derivatives and distinct within-diet lipid patterns, while KEGG enrichment provided corrected statistical support only for aromatic amino-acid biosynthesis among B+S increases. Together, the data support free amino acids as a priority axis for acute heat-stress biomarker development and nominate lipid remodeling and oxidative-load chemistry for targeted follow-up. Validation with authentic standards, a graded temperature/time series, and an integrated factorial analysis will be required before these signals can be used as mechanistic or field-diagnostic markers.

## Figures and Tables

**Table 1:** Selected metabolites significantly altered in B+S workers after acute heat exposure (45 °C, 3 h) relative to the 37 °C control. Body untargeted UPLC-QTOF/MS profiling integrated ESI+ and ESI− data from workers fed bee bread plus sucrose (B+S). Twenty-two metabolites with higher abundance and 18 with lower abundance at 45 °C are shown; the complete list of 149 significant metabolites is provided in Table S1.

Super Class	Compound	Neutral Mass	RT (min)	KEGG ID	ESI	*p*	*p* (Corr)	Log FC (45 vs. 37)
Up-regulated at 45 °C ↑ (22 selected of 78)								
Amino Acids and Peptides	Sarcosine	89.0476	4.37	C00213	ESI−	3.1 × 10^−9^	1.8 × 10^−6^	17.92
	4,6-Dihydroxyquinoline	161.0477	1.53	C05639	ESI−	7.3 × 10^−4^	1.6 × 10^−2^	2.75
	Phenylpyruvic acid	164.0473	6.06	C00166	ESI+	5.0 × 10^−5^	1.9 × 10^−3^	2.52
	L-Isoleucine	131.0948	4.39	C00407	ESI+	1.4 × 10^−3^	4.5 × 10^−2^	2.15
	L-Phenylalanine	165.0788	2.04	C00079	ESI−	2.2 × 10^−3^	4.3 × 10^−2^	2.05
	DL-Serine	105.0425	4.97	C00716	ESI−	2.4 × 10^−4^	5.6 × 10^−3^	1.38
	L-Glutamate	147.0532	6.91	C00025	ESI−	5.1 × 10^−4^	1.1 × 10^−2^	1.00
Amines and Polyamines	Piperidine	85.0892	4.39	C01746	ESI+	7.9 × 10^−4^	2.6 × 10^−2^	2.48
Organic Acids	Maleic acid	116.0109	7.46	C01384	ESI−	2.1 × 10^−5^	5.9 × 10^−4^	20.87
Carbohydrates and Sugars	N-Acetyl-D-fucosamine	205.0953	8.85	C15480	ESI+	3.7 × 10^−5^	1.4 × 10^−3^	2.50
	D-Sorbitol	182.0787	4.91	C00794	ESI−	3.9 × 10^−4^	9.0 × 10^−3^	1.07
Nucleotides and Nucleosides	Deoxyadenosine monophosphate	331.0669	1.56	C00360	ESI−	2.6 × 10^−7^	1.7 × 10^−5^	18.03
	Inosine 5′-monophosphate (IMP)	348.0475	9.34	C00130	ESI+	1.7 × 10^−6^	1.3 × 10^−4^	13.92
Vitamins and Cofactors	lipoamide	205.0587	7.49	C00248	ESI−	1.1 × 10^−5^	3.4 × 10^−4^	17.51
	Pyridoxine (Vitamin B6)	169.0740	1.81	C00314	ESI+	5.6 × 10^−6^	2.9 × 10^−4^	16.69
	d-Dethiobiotin	214.1327	2.11	C01909	ESI−	2.2 × 10^−3^	4.4 × 10^−2^	2.38
Fatty Acyls	2,3-Dinor-6-keto-PGF1α	342.2028	1.09	—	ESI−	4.4 × 10^−4^	9.9 × 10^−3^	18.91
	4-hydroxy enanthoic acid	146.0936	1.43	—	ESI−	1.0 × 10^−6^	4.6 × 10^−5^	17.81
Sphingolipids	Dehydrophytosphingosine	315.2779	0.82	—	ESI+	8.2 × 10^−6^	4.0 × 10^−4^	14.82
Sterols and Steroids	2-deoxyecdysone 22-phosphate	528.2839	5.41	—	ESI+	3.8 × 10^−5^	1.5 × 10^−3^	1.06
Terpenoids	Geranylfarnesyl diphosphate	518.2556	0.98	C17435	ESI−	2.6 × 10^−8^	4.2 × 10^−6^	17.47
Plant-derived Metabolites	Linamarin	247.1058	9.33	C01594	ESI+	2.4 × 10^−4^	8.5 × 10^−3^	3.34
Down-regulated at 45 °C ↓ (18 selected of 71)								
Amino Acids and Peptides	(2S)-2-{[1-(R)-Carboxyethyl]amino}pentanoate	189.1002	3.51	C06326	ESI+	2.5 × 10^−7^	8.6 × 10^−5^	−16.88
	D-Ornithine	132.0900	12.88	C00515	ESI+	5.4 × 10^−7^	9.3 × 10^−5^	−16.56
Amines and Polyamines	N-Acetylputrescine	130.1105	9.04	C02714	ESI+	8.9 × 10^−7^	1.0 × 10^−4^	−16.43
Organic Acids	3-Ethylmalate	162.0523	1.59	C01989	ESI−	9.6 × 10^−9^	3.0 × 10^−6^	−24.18
	(Z)-But-1-ene-1,2,4-tricarboxylate	188.0328	2.16	C04002	ESI−	7.1 × 10^−6^	2.3 × 10^−4^	−16.47
Carbohydrates and Sugars	2-Amino-3,7-dideoxy-D-threo-hept-6-ulosonic acid	191.0791	5.29	C16850	ESI−	6.7 × 10^−6^	2.2 × 10^−4^	−19.47
	N,N′-Diacetylchitobiosyldiphosphodolichol	780.2500	4.80	C04537	ESI−	1.8 × 10^−7^	1.3 × 10^−5^	−16.55
Vitamins and Cofactors	Precorrin 4	908.3338	4.83	C06407	ESI−	6.4 × 10^−8^	7.5 × 10^−6^	−16.14
Fatty Acyls	13-methyl-hexadecanoic acid	270.2556	0.82	—	ESI−	7.8 × 10^−7^	3.8 × 10^−5^	−17.98
	13,14-dihydro-15-keto-tetranor PGE2	298.1776	1.75	—	ESI−	1.2 × 10^−4^	3.0 × 10^−3^	−17.70
	epi-4′-hydroxyjasmonic acid	226.1199	1.19	—	ESI+	1.3 × 10^−5^	5.8 × 10^−4^	−17.59
Glycerolipids	Tributyrin	302.1725	3.44	C13870	ESI−	1.9 × 10^−5^	5.5 × 10^−4^	−16.73
	DG(18:0/18:0/0:0)	624.5699	1.31	—	ESI+	1.2 × 10^−5^	5.4 × 10^−4^	−15.35
	1,2-Dihexanoyl-sn-glycerol	288.1933	6.27	—	ESI−	1.0 × 10^−7^	9.1 × 10^−6^	−15.08
Phospholipids	PE(O-18:1(9Z)/0:0)	465.3225	2.44	—	ESI+	3.6 × 10^−5^	1.4 × 10^−3^	−17.78
	PE(18:0(10(R)Me)/16:0)	733.5634	1.03	—	ESI−	1.9 × 10^−7^	1.4 × 10^−5^	−16.29
	CL(1′-[16:0/18:1(11Z)],3′-[16:0/18:1(11Z)])	1405.0021	0.88	—	ESI+	4.0 × 10^−6^	2.2 × 10^−4^	−15.22
Sphingolipids	Glucosylsphingosine	461.3335	1.38	C03108	ESI−	1.2 × 10^−6^	5.4 × 10^−5^	−17.67

Neutral mass, monoisotopic mass of the neutral molecule; RT, retention time; ESI, ionization mode; *p*, unadjusted *t*-test *p* value; *p* (Corr), Benjamini–Hochberg-adjusted *p* value; Log FC, log_2_ fold change (45 °C vs. 37 °C). Compound annotations are putative MSI level 2–3 assignments based on accurate mass, isotope pattern, and database matching and were not confirmed with authentic standards. Em dash (—) indicates unavailable data. Very large absolute log_2_ FC values reflect features detected predominantly in one temperature group and should not be interpreted as precise abundance ratios.

**Table 2:** Selected metabolites significantly altered in S workers after acute heat exposure (45 °C, 3 h) relative to the 37 °C control. Body untargeted UPLC-QTOF/MS profiling integrated ESI+ and ESI− data from workers fed sucrose alone (S). Twenty metabolites with higher abundance and 20 with lower abundance at 45 °C are shown; the complete list of 123 significant metabolites is provided in Table S2.

Super Class	Compound	Neutral Mass	RT (min)	KEGG ID	ESI	*p*	*p* (Corr)	Log FC (45 vs. 37)
Up-regulated at 45 °C ↑ (20 selected of 56)								
Amino Acids and Peptides	N-Acetyl-L-Histidine	197.0791	3.02	C02997	ESI−	1.5 × 10^−6^	7.6 × 10^−5^	19.06
	N-Pyruvoyl-5-methoxy-3-hydroxyanthranilate	253.0590	2.93	C11467	ESI−	3.0 × 10^−6^	1.3 × 10^−4^	16.77
	Quisqualic acid	189.0392	5.46	C08296	ESI+	1.9 × 10^−7^	3.1 × 10^−5^	15.33
	L-Dopa	197.0685	7.90	C00355	ESI−	1.7 × 10^−6^	8.5 × 10^−5^	15.03
	Prephenic acid	226.0482	5.39	C00254	ESI+	6.1 × 10^−6^	3.0 × 10^−4^	13.73
Amines and Polyamines	N4-Phosphoagmatine	210.0882	11.57	C02726	ESI−	8.3 × 10^−6^	2.7 × 10^−4^	16.56
Organic Acids	Mesaconic acid	130.0264	10.95	C01732	ESI−	3.5 × 10^−7^	4.1 × 10^−5^	20.48
	Aconitic acid	174.0165	10.98	C00417	ESI−	5.3 × 10^−6^	1.8 × 10^−4^	18.65
Carbohydrates and Sugars	D-Mannonate	196.0580	2.51	C00514	ESI−	3.4 × 10^−6^	1.3 × 10^−4^	21.16
	1,5-Anhydro-4-deoxy-D-glycero-hex-1-en-3-ulose	144.0423	7.81	C19826	ESI−	2.9 × 10^−9^	1.4 × 10^−5^	20.74
	Levan	504.1666	5.59	C01355	ESI−	1.8 × 10^−5^	5.2 × 10^−4^	15.91
Nucleotides and Nucleosides	1-Methyladenine	149.0703	1.53	C02216	ESI+	2.9 × 10^−5^	1.1 × 10^−3^	20.78
Fatty Acyls	11-methyl-tridecanoic acid	228.2089	1.29	—	ESI−	2.2 × 10^−7^	3.4 × 10^−5^	20.70
	Δ17-6-keto-PGF1α	368.2198	1.83	—	ESI−	7.2 × 10^−7^	5.1 × 10^−5^	17.25
	8,9-DiHETrE-EA	381.2864	1.07	—	ESI−	4.6 × 10^−7^	4.5 × 10^−5^	17.07
Glycerolipids	MGDG(18:5(3Z,6Z,9Z,12Z,15Z)/18:4(6Z,9Z,12Z,15Z))	768.4812	0.96	—	ESI−	5.7 × 10^−6^	2.0 × 10^−4^	14.64
Phospholipids	PI(O-18:0/18:3(6Z,9Z,12Z))	846.5643	1.30	—	ESI−	2.8 × 10^−7^	3.7 × 10^−5^	18.75
	PE(8:0/8:0)	467.2666	1.44	—	ESI−	1.7 × 10^−5^	5.0 × 10^−4^	16.50
Sphingolipids	D-erythro-Sphingosine C-15	257.2348	1.52	—	ESI+	1.9 × 10^−6^	1.3 × 10^−4^	15.17
Terpenoids	(10S)-Juvenile hormone III diol	284.1987	1.39	C16505	ESI−	7.9 × 10^−5^	2.1 × 10^−3^	22.30
Down-regulated at 45 °C ↓ (20 selected of 67)								
Amino Acids and Peptides	5-Aminopentanoic acid	117.0790	1.42	C00431	ESI+	7.4 × 10^−8^	1.7 × 10^−5^	−18.98
	Kynurenine	208.0864	2.59	C00328	ESI+	4.7 × 10^−5^	1.7 × 10^−3^	−17.00
	Melatonin	232.1201	8.49	C01598	ESI+	5.9 × 10^−6^	2.9 × 10^−4^	−14.55
Amines and Polyamines	1-Methylhistamine	125.0952	8.32	C05127	ESI+	6.6 × 10^−5^	2.3 × 10^−3^	−17.16
	Histamine	111.0795	4.56	C00388	ESI−	4.7 × 10^−6^	1.7 × 10^−4^	−14.36
Organic Acids	(S)-Acetoin	88.0523	4.29	C01769	ESI−	6.5 × 10^−7^	4.9 × 10^−5^	−21.62
	2-Methyleneglutarate	144.0421	7.80	C02930	ESI−	1.8 × 10^−7^	3.2 × 10^−5^	−19.28
Carbohydrates and Sugars	4-(4-Deoxy-β-D-gluc-4-enuronosyl)-D-galacturonate	352.0639	1.62	C06118	ESI−	9.8 × 10^−8^	2.6 × 10^−5^	−20.95
	1,4-beta-D-Glucan	536.1591	3.99	C00760	ESI−	8.9 × 10^−6^	2.9 × 10^−4^	−20.87
	L-Galactono-1,4-lactone	178.0480	1.89	C01115	ESI−	2.6 × 10^−7^	3.7 × 10^−5^	−18.09
Nucleotides and Nucleosides	dIMP	332.0534	1.56	C06196	ESI−	1.4 × 10^−4^	3.4 × 10^−3^	−20.65
Vitamins and Cofactors	Bilirubin glucuronide	760.2974	1.44	C03033	ESI−	1.4 × 10^−3^	3.1 × 10^−2^	−14.08
Fatty Acyls	13,14-Dihydro-15-keto-tetranor-PGF1α	300.1931	2.55	—	ESI−	1.7 × 10^−5^	5.0 × 10^−4^	−19.08
	4Z-decenoic acid	170.1308	1.20	—	ESI+	1.7 × 10^−8^	1.1 × 10^−5^	−17.51
	Tricosanyl oleate	576.5823	0.99	—	ESI−	7.3 × 10^−7^	5.1 × 10^−5^	−17.05
Phospholipids	PE(19:0/0:0)	495.3331	1.30	—	ESI+	1.4 × 10^−5^	6.0 × 10^−4^	−18.22
	PI(20:1(11Z)/14:0)	836.5395	1.03	—	ESI−	4.1 × 10^−4^	9.3 × 10^−3^	−17.25
	PI(18:3(9Z,12Z,15Z)/0:0)	594.2807	4.42	—	ESI+	7.5 × 10^−4^	2.4 × 10^−2^	−1.55
Sphingolipids	Dehydrophytosphingosine	315.2778	1.78	—	ESI+	9.6 × 10^−7^	8.1 × 10^−5^	−22.27
Sterols and Steroids	Δ8,14-Sterol	410.3536	3.95	C11508	ESI+	1.7 × 10^−6^	1.3 × 10^−4^	−14.78

Neutral mass, monoisotopic mass of the neutral molecule; RT, retention time; ESI, ionization mode; p, unadjusted *t*-test *p* value; *p* (Corr), Benjamini–Hochberg-adjusted *p* value; Log FC, log_2_ fold change (45 °C vs. 37 °C). Compound annotations are putative MSI level 2–3 assignments based on accurate mass, isotope pattern, and database matching and were not confirmed with authentic standards. Em dash (—) indicates unavailable data. Very large absolute log_2_ FC values reflect features detected predominantly in one temperature group and should not be interpreted as precise abundance ratios.

## Data Availability

The data presented in this study are available upon request from the corresponding authors.

## Supplementary Materials

The following supporting information can be downloaded at: https://www.mdpi.com/article/10.3390/ijms27209237/s1.

## Abbreviations

The following abbreviations are used in this manuscript:

B+S	Bee bread plus sucrose diet
BH	Benjamini–Hochberg
ESI+	Positive electrospray ionization mode
ESI−	Negative electrospray ionization mode
FC	Fold change
FDR	False discovery rate
HILIC	Hydrophilic interaction liquid chromatography
HMDB	Human Metabolome Database
JH	Juvenile hormone
KEGG	Kyoto Encyclopedia of Genes and Genomes
LC-MS	Liquid chromatography–mass spectrometry
LC-QTOF/MS	Liquid chromatography–quadrupole time-of-flight mass spectrometry
Leu/Ile	Unresolved leucine/isoleucine signal
LIPID MAPS	Lipid Metabolites and Pathways Strategy
MPP	Mass Profiler Professional
MS	Mass spectrometry
MSI	Metabolomics Standards Initiative
m/z	Mass-to-charge ratio
OECD	Organization for Economic Co-operation and Development
PA	Phosphatidic acid
PCA	Principal component analysis
PI	Phosphatidylinositol
PS	Phosphatidylserine
RH	Relative humidity
ROS	Reactive oxygen species
RT	Retention time
S	Sucrose-only diet
TCA	Tricarboxylic acid
UHPLC	Ultra-high-performance liquid chromatography
UPLC-QTOF/MS	Ultra-performance liquid chromatography–quadrupole time-of-flight mass spectrometry
