# Supplementary material for: Metabolomic Profiling Reveals Heat-Responsive Metabolic Markers in Honey Bee Workers Under Contrasting Diets
Source: Int J Mol Sci. 2026 Oct 17;27(20):9237. doi: 10.3390/ijms27209237 (PMC13705331; doi:10.3390/ijms27209237)
Supplement: Supplementary file 1 [file ijms-27-09237-s001.zip › Supplementary Figure S1-legend-업로드 시 사용.pdf]

Supplementary Figure S1. Additional representative heat-responsive metabolites quantified in every sample of both temperature groups. Box-and-point plots show log2 abundance at 37 °C (blue) and 45 °C (terracotta) for three biological replicates per temperature. The B+S metabolites shown are L-phenylalanine, phenylpyruvic acid, D-sorbitol, N-acetyl-D-fucosamine, piperidine, d-dethiobiotin, 2-deoxyecdysone 22-phosphate, leucopterin, D-(+)-cellobiose, and isorhamnetin 3-malonylglucoside, whereas PI(18:3/0:0) and nitiducarpin are from the S dataset. Each panel displays the Benjamini–Hochberg-adjusted p value (q) from the two-group t-test implemented in MPP for the comparison of 45 °C versus 37 °C. Asterisks indicate $q < 0.05$ (*) or $q < 0.01$ (**).
