# Supplementary material for: Metabolomic Profiling Reveals Heat-Responsive Metabolic Markers in Honey Bee Workers Under Contrasting Diets
Source: Int J Mol Sci. 2026 Oct 17;27(20):9237. doi: 10.3390/ijms27209237 (PMC13705331; doi:10.3390/ijms27209237)
Supplement: Supplementary file 1 [file ijms-27-09237-s001.zip › TableS1_BS_37vs45.pdf]

Table S1. Complete list of metabolites significantly altered in B+S workers after heat exposure (45 °C, 3 h) relative to the 37 °C control.

All 149 named metabolites passing $|FC| \geq 2$ and Benjamini–Hochberg adjusted $p < 0.05$ in the B+S (bee bread + sucrose) diet group, ordered by direction of regulation, super class, and $|\log_2 FC|$.

Super Class	Compound	Formula	Neutral mass	RT (min)	KEGG ID	ESI	p	p (Corr)	Log FC (45 vs 37)	FC (abs)
**Up-regulated at 45 °C ↑ (n = 78)**										
**Amino Acids & Peptides**	2S-aminoheptanoic acid	C7 H15 N O2	145.1101	1.79	—	ESI-	1.2×10^{-4}	2.9×10^{-3}	19.33	6.6×10^{5}
	Methyl N-butyrylglycine	C7 H13 N O3	159.0892	2.48	—	ESI-	9.6×10^{-7}	4.5×10^{-5}	19.22	6.1×10^{5}
	DL-2-Aminoadipic acid	C6 H11 N O4	161.0689	8.74	—	ESI+	2.0×10^{-7}	8.6×10^{-5}	18.25	3.1×10^{5}
	Sarcosine	C3 H7 N O2	89.0476	4.37	C00213	ESI-	3.1×10^{-9}	1.8×10^{-6}	17.92	2.5×10^{5}
	VPGPR Enterostatin	C23 H40 N8 O6	524.3064	1.04	—	ESI-	7.2×10^{-6}	2.3×10^{-4}	16.76	1.1×10^{5}
	Aminoacetone	C3 H7 N O	73.0529	7.98	C01888	ESI+	5.2×10^{-7}	9.1×10^{-5}	16.43	8.8×10^{4}
	Acetyl-L-Cysteine	C5 H9 N O3 S	163.0303	2.79	C06809	ESI-	1.1×10^{-7}	9.5×10^{-6}	16.35	8.4×10^{4}
	Cysteine-Homocysteine disulfide	C7 H14 N2 O4 S2	254.0394	3.03	—	ESI-	9.5×10^{-11}	2.7×10^{-7}	15.66	5.2×10^{4}
	Thiomorpholine 3-carboxylate	C5 H9 N O2 S	147.0356	8.30	C03901	ESI+	5.1×10^{-7}	9.1×10^{-5}	15.57	4.9×10^{4}
	4,6-Dihydroxyquinoline	C9 H7 N O2	161.0477	1.53	C05639	ESI-	7.3×10^{-4}	1.6×10^{-2}	2.75	6.7
	Phenylpyruvic acid	C9 H8 O3	164.0473	6.06	C00166	ESI+	5.0×10^{-5}	1.9×10^{-3}	2.52	5.7
	L-Isoleucine	C6 H13 N O2	131.0948	4.39	C00407	ESI+	1.4×10^{-3}	4.5×10^{-2}	2.15	4.4
	L-Phenylalanine	C9 H11 N O2	165.0788	2.04	C00079	ESI-	2.2×10^{-3}	4.3×10^{-2}	2.05	4.1
	DL-Serine	C3 H7 N O3	105.0425	4.97	C00716	ESI-	2.4×10^{-4}	5.6×10^{-3}	1.38	2.6
	L-Glutamate	C5 H9 N O4	147.0532	6.91	C00025	ESI-	5.1×10^{-4}	1.1×10^{-2}	1.00	2.0
**Amines & Polyamines**	N1,N5-Tri-di(hydroxyferuloyl)-N10-sinapoyl-spermidine	C38 H45 N3 O12	735.3006	2.93	C18073	ESI-	9.2×10^{-6}	2.9×10^{-4}	16.10	7.0×10^{4}
	Piperidine	C5 H11 N	85.0892	4.39	C01746	ESI+	7.9×10^{-4}	2.6×10^{-2}	2.48	5.6
**Organic Acids**	Maleic acid	C4 H4 O4	116.0109	7.46	C01384	ESI-	2.1×10^{-5}	5.9×10^{-4}	20.87	1.9×10^{6}
	2-Hydroxy-3-chloropenta-2,4-dienoate	C5 H5 Cl O3	147.9921	1.77	C11353	ESI-	3.8×10^{-9}	1.9×10^{-6}	18.57	3.9×10^{5}

Super Class	Compound	Formula	Neutral mass	RT (min)	KEGG ID	ESI	p	p (Corr)	Log FC (45 vs 37)	FC (abs)
	2-(Hydroxymethyl)-3-(acetamidomethylene)succinate	C8 H11 N O6	217.0586	6.89	C04690	ESI-	2.2×10^{-8}	4.0×10^{-6}	17.29	1.6×10^{5}
	2,5-Dichloro-4-oxohex-2-enedioate	C6 H4 Cl2 O5	225.9441	7.34	C12835	ESI+	3.0×10^{-6}	1.9×10^{-4}	16.51	9.3×10^{4}
**Carbohydrates & Sugars**	2-(alpha-D-Galactosyl)-sn-glycerol 3-phosphate	C9 H19 O11 P	334.0663	4.97	C04641	ESI-	7.5×10^{-7}	3.7×10^{-5}	17.21	1.5×10^{5}
	UDP-MurNAc(oyl-L-Ala-D-γ-Glu-L-Lys-D-Ala-D-Ala)	C40 H65 N9 O26 P2	1149.3538	12.08	C04702	ESI-	2.6×10^{-9}	1.7×10^{-6}	16.73	1.1×10^{5}
	2-(acetylamino)-1,5-anhydro-2-deoxy-3-O-b-D-galactopyranosyl-D-arabino-Hex-1-enitol	C14 H23 N O10	365.1324	5.37	—	ESI+	8.5×10^{-7}	9.9×10^{-5}	14.52	2.4×10^{4}
	GDP-4-Dehydro-6-deoxy-D-mannose	C16 H23 N5 O15 P2	587.0663	7.59	C01222	ESI+	6.8×10^{-7}	9.9×10^{-5}	13.41	1.1×10^{4}
	N-Acetyl-D-fucosamine	C8 H15 N O5	205.0953	8.85	C15480	ESI+	3.7×10^{-5}	1.4×10^{-3}	2.50	5.7
	D-Sorbitol	C6 H14 O6	182.0787	4.91	C00794	ESI-	3.9×10^{-4}	9.0×10^{-3}	1.07	2.1
**Nucleotides & Nucleosides**	Deoxyadenosine monophosphate	C10 H14 N5 O6 P	331.0669	1.56	C00360	ESI-	2.6×10^{-7}	1.7×10^{-5}	18.03	2.7×10^{5}
	8-hydroxy Guanosine	C10 H13 N5 O6	299.0876	2.19	—	ESI-	3.1×10^{-6}	1.1×10^{-4}	17.71	2.1×10^{5}
	Urate D-ribonucleotide	C10 H13 N4 O10 P	380.0369	9.47	C03312	ESI-	6.9×10^{-6}	2.3×10^{-4}	14.67	2.6×10^{4}
	5'-Oxoinosine	C10 H10 N4 O5	266.0648	11.41	C01993	ESI+	1.1×10^{-6}	1.1×10^{-4}	13.93	1.6×10^{4}
	Inosine 5'-monophosphate (IMP)	C10 H13 N4 O8 P	348.0475	9.34	C00130	ESI+	1.7×10^{-6}	1.3×10^{-4}	13.92	1.5×10^{4}
**Vitamins & Cofactors**	Deazaflavin	C11 H7 N3 O2	213.0517	4.95	C01800	ESI-	3.3×10^{-7}	2.1×10^{-5}	19.77	8.9×10^{5}
	dexpanthenol	C9 H19 N O4	205.1309	4.82	C05944	ESI-	5.5×10^{-9}	2.4×10^{-6}	17.54	1.9×10^{5}
	lipoamide	C8 H15 N O S2	205.0587	7.49	C00248	ESI-	1.1×10^{-5}	3.4×10^{-4}	17.51	1.9×10^{5}
	Pyridoxine (Vitamin B6)	C8 H11 N O3	169.0740	1.81	C00314	ESI+	5.6×10^{-6}	2.9×10^{-4}	16.69	1.1×10^{5}
	Reduced coenzyme F420	C29 H38 N5 O18 P	775.1923	9.35	C01080	ESI-	3.9×10^{-11}	2.7×10^{-7}	16.46	9.0×10^{4}
	1α,24,25,28-Tetrahydroxyvitamin D2	C28 H44 O5	460.3194	2.01	—	ESI+	2.1×10^{-5}	8.8×10^{-4}	14.83	2.9×10^{4}
	d-Dethiobiotin	C10 H18 N2 O3	214.1327	2.11	C01909	ESI-	2.2×10^{-3}	4.4×10^{-2}	2.38	5.2

Super Class	Compound	Formula	Neutral mass	RT (min)	KEGG ID	ESI	p	p (Corr)	Log FC (45 vs 37)	FC (abs)
**Fatty Acyls**	2,3-Dinor-6-keto-PGF1α	C18 H30 O6	342.2028	1.09	—	ESI-	4.4×10^{-4}	9.9×10^{-3}	18.91	4.9×10^{5}
	4-hydroxy enanthoic acid	C7 H14 O3	146.0936	1.43	—	ESI-	1.0×10^{-6}	4.6×10^{-5}	17.81	2.3×10^{5}
	Sativic acid	C18 H36 O6	348.2514	1.78	—	ESI+	2.6×10^{-6}	1.7×10^{-4}	17.36	1.7×10^{5}
	Leukotriene D4	C25 H40 N2 O6 S	496.2631	0.96	C05951	ESI-	1.6×10^{-8}	3.6×10^{-6}	16.40	8.6×10^{4}
	N-arachidonoyl dihydroxypropylamine	C23 H39 N O3	377.2972	1.60	—	ESI+	2.2×10^{-6}	1.5×10^{-4}	15.67	5.2×10^{4}
	2,4,6-trimethyl-2,15-tetracosadienoic acid	C27 H50 O2	406.3797	4.80	—	ESI+	6.6×10^{-7}	9.9×10^{-5}	14.48	2.3×10^{4}
**Glycerolipids**	DG(22:0/20:4(5Z,8Z,11Z,14Z)/0:0)	C45 H80 O5	700.5928	1.04	—	ESI-	1.4×10^{-8}	3.4×10^{-6}	16.66	1.0×10^{5}
**Phospholipids**	Phosphatidyl glycerol	C6 H15 O8 P	246.0503	4.46	C03274	ESI-	1.7×10^{-6}	7.3×10^{-5}	16.84	1.2×10^{5}
	PG(18:4(6Z,9Z,12Z,15Z)/14:1(9Z))	C38 H65 O10 P	712.4338	1.03	—	ESI-	8.4×10^{-8}	8.3×10^{-6}	16.48	9.1×10^{4}
	PA(20:1(11Z)/12:0)	C35 H67 O8 P	646.4590	1.44	—	ESI-	1.0×10^{-6}	4.6×10^{-5}	16.02	6.7×10^{4}
	PE(20:4(5Z,8Z,11Z,14Z)/14:0)	C39 H70 N O8 P	711.4873	1.03	C00350	ESI-	2.5×10^{-5}	6.9×10^{-4}	16.02	6.6×10^{4}
	1-(8-[3]-ladderane-octanoyl)-2-(8-[3]-ladderane-octanyl)-sn-glycero-3-phospho-(1'-sn-glycerol)	C46 H77 O9 P	804.5283	4.71	—	ESI-	4.9×10^{-4}	1.1×10^{-2}	1.19	2.3
**Sphingolipids**	Dehydrophytosphingosine	C18 H37 N O3	315.2779	0.82	—	ESI+	8.2×10^{-6}	4.0×10^{-4}	14.82	2.9×10^{4}
	KDNalpha2-3Galbeta1-4Glcbeta-Cer(d18:1/26:1(17Z))	C65 H119 N O21	1249.8310	6.59	—	ESI+	1.6×10^{-6}	1.3×10^{-4}	14.17	1.8×10^{4}
**Sterols & Steroids**	7α,12α-Dihydroxy-5β-cholan-24-oic acid	C24 H40 O4	392.2926	8.14	—	ESI+	3.2×10^{-6}	1.9×10^{-4}	15.49	4.6×10^{4}
	Convalloside	C35 H52 O15	712.3288	0.98	C19986	ESI-	5.5×10^{-6}	1.9×10^{-4}	15.33	4.1×10^{4}
	A-Nor-5alpha-cholestan-2-one	C26 H44 O	372.3383	6.08	C15292	ESI+	2.7×10^{-6}	1.8×10^{-4}	13.79	1.4×10^{4}
	2-deoxyecdysone 22-phosphate	C27 H45 O8 P	528.2839	5.41	—	ESI+	3.8×10^{-5}	1.5×10^{-3}	1.06	2.1
**Terpenoids**	Geranylfarnesyl diphosphate	C25 H44 O7 P2	518.2556	0.98	C17435	ESI-	2.6×10^{-8}	4.2×10^{-6}	17.47	1.8×10^{5}
	dolichyl phosphate	C25 H45 O4 P	440.3063	5.44	C00110	ESI+	4.8×10^{-6}	2.5×10^{-4}	15.64	5.1×10^{4}
	Carnosifloside I	C42 H68 O13	780.4646	1.29	C08790	ESI-	1.1×10^{-7}	9.8×10^{-6}	15.27	3.9×10^{4}
**Phenolic Compounds**	Gossypetin	C15 H10 O8	318.0362	1.58	C04109	ESI-	6.8×10^{-5}	1.7×10^{-3}	19.28	6.4×10^{5}
	2,4,4'-Trihydroxydihydrochalcone	C15 H14 O4	258.0946	2.49	—	ESI-	3.6×10^{-7}	2.2×10^{-5}	18.35	3.3×10^{5}

Super Class	Compound	Formula	Neutral mass	RT (min)	KEGG ID	ESI	p	p (Corr)	Log FC (45 vs 37)	FC (abs)
	Munetone	C26 H24 O5	416.1629	1.92	—	ESI-	3.2×10^{-7}	2.1×10^{-5}	16.84	1.2×10^{5}
	Luteolin 7,3'-dimethyl ether 4'-glucoside	C23 H24 O11	476.1327	1.48	—	ESI-	1.1×10^{-5}	3.3×10^{-4}	16.71	1.1×10^{5}
	Isorhamnetin 3-(4''-sulfatorutinoside)	C28 H32 O19 S	704.1248	6.39	—	ESI+	8.2×10^{-5}	3.1×10^{-3}	15.46	4.5×10^{4}
	Prim-o-glucosylcimifugin	C22 H28 O11	468.1614	6.02	—	ESI+	9.3×10^{-7}	1.0×10^{-4}	15.19	3.7×10^{4}
	Luteolin 4'-methyl ether 7-(6''-malonylglucoside)	C25 H24 O14	548.1173	4.83	—	ESI-	4.2×10^{-6}	1.5×10^{-4}	14.77	2.8×10^{4}
	Ikarisoside D	C28 H30 O11	542.1763	5.61	—	ESI+	1.8×10^{-5}	7.5×10^{-4}	14.75	2.8×10^{4}
	Anthraquinone	C14 H8 O2	208.0523	11.25	C16207	ESI+	2.6×10^{-6}	1.7×10^{-4}	13.25	9,761.0
	Quercetin 3-(2'''-galloylglucosyl)-(1-2)-alpha-L-arabinofuranoside	C33 H32 O20	748.1498	5.85	—	ESI+	1.0×10^{-6}	1.1×10^{-4}	13.06	8,525.2
	Isoorientin 2''-p-hydroxybenzoate	C28 H24 O13	568.1238	5.65	—	ESI+	1.3×10^{-6}	1.2×10^{-4}	13.00	8,200.6
	Dichamanetin	C29 H24 O6	468.1593	2.86	—	ESI-	6.8×10^{-5}	1.7×10^{-3}	2.52	5.7
**Alkaloids**	Hymenocardine	C37 H50 N6 O6	674.3799	0.97	C10004	ESI-	2.1×10^{-6}	8.3×10^{-5}	15.38	4.3×10^{4}
	Vomicine	C22 H24 N2 O4	380.1736	1.25	C09255	ESI+	4.1×10^{-5}	1.6×10^{-3}	15.32	4.1×10^{4}
**Plant-derived Metabolites**	3-Methylbutyraldehyde oxime	C5 H11 N O	101.0842	11.42	C17255	ESI+	8.3×10^{-7}	9.9×10^{-5}	16.09	7.0×10^{4}
	Linoside A	C32 H38 O16	678.2149	9.49	—	ESI+	8.9×10^{-7}	1.0×10^{-4}	13.83	1.5×10^{4}
	Indican	C14 H17 N O6	295.1044	4.01	C08481	ESI+	1.4×10^{-3}	4.5×10^{-2}	13.26	9,805.4
	Linamarin	C10 H17 N O6	247.1058	9.33	C01594	ESI+	2.4×10^{-4}	8.5×10^{-3}	3.34	10.1
**Down-regulated at 45 °C ↓ (n = 71)**										
**Amino Acids & Peptides**	4,6-Dihydroxyquinoline	C9 H7 N O2	161.0477	1.19	C05639	ESI+	3.4×10^{-7}	8.8×10^{-5}	-18.09	2.8×10^{5}
	?-Valerolactam	C5 H9 N O	99.0682	1.20	—	ESI+	9.4×10^{-8}	8.6×10^{-5}	-17.87	2.4×10^{5}
	Cucumopine	C11 H13 N3 O6	283.0818	1.91	C08475	ESI-	6.8×10^{-8}	7.8×10^{-6}	-17.65	2.1×10^{5}
	(2S)-2-{[1-(R)-Carboxyethyl]amino}pentanoate	C8 H15 N O4	189.1002	3.51	C06326	ESI+	2.5×10^{-7}	8.6×10^{-5}	-16.88	1.2×10^{5}
	D-Ornithine	C5 H12 N2 O2	132.0900	12.88	C00515	ESI+	5.4×10^{-7}	9.3×10^{-5}	-16.56	9.6×10^{4}
	Leucine Enkephalin	C28 H37 N5 O7	555.2696	1.04	—	ESI-	7.6×10^{-8}	8.1×10^{-6}	-16.51	9.4×10^{4}

Super Class	Compound	Formula	Neutral mass	RT (min)	KEGG ID	ESI	p	p (Corr)	Log FC (45 vs 37)	FC (abs)
	Methyl N-butyrylglycine	C7 H13 N O3	159.0890	4.19	—	ESI+	1.1×10^{-6}	1.1×10^{-4}	-16.16	7.3×10^{4}
	Formylmethionyl-leucyl-phenylalanine methyl ester	C22 H33 N3 O5 S	451.2132	1.54	C11221	ESI-	9.5×10^{-6}	2.9×10^{-4}	-15.76	5.6×10^{4}
**Amines & Polyamines**	N-Acetylputrescine	C6 H14 N2 O	130.1105	9.04	C02714	ESI+	8.9×10^{-7}	1.0×10^{-4}	-16.43	8.8×10^{4}
	Philanthotoxin 343	C23 H41 N5 O3	435.3194	9.70	C13806	ESI+	1.1×10^{-5}	5.2×10^{-4}	-13.78	1.4×10^{4}
**Organic Acids**	3-Ethylmalate	C6 H10 O5	162.0523	1.59	C01989	ESI-	9.6×10^{-9}	3.0×10^{-6}	-24.18	1.9×10^{7}
	4-hydroxy-valeric acid	C5 H10 O3	118.0627	1.51	—	ESI-	4.8×10^{-7}	2.7×10^{-5}	-23.21	9.7×10^{6}
	(Z)-But-1-ene-1,2,4-tricarboxylate	C7 H8 O6	188.0328	2.16	C04002	ESI-	7.1×10^{-6}	2.3×10^{-4}	-16.47	9.0×10^{4}
	4-Deoxytetronic acid	C4 H6 O2	86.0369	1.77	C01770	ESI+	7.6×10^{-7}	9.9×10^{-5}	-14.81	2.9×10^{4}
	2-Oxo-3-hydroxy-4-phosphobutanoate	C4 H7 O8 P	213.9880	3.88	C06054	ESI+	5.2×10^{-4}	1.8×10^{-2}	-13.01	8,275.4
**Carbohydrates & Sugars**	N-Acetyl-D-fucosamine	C8 H15 N O5	205.0952	8.71	C15480	ESI+	1.9×10^{-8}	8.6×10^{-5}	-24.71	2.8×10^{7}
	2-Amino-3,7-dideoxy-D-threo-hept-6-ulosonic acid	C7 H13 N O5	191.0791	5.29	C16850	ESI-	6.7×10^{-6}	2.2×10^{-4}	-19.47	7.3×10^{5}
	Streptobiosamine	C13 H23 N O9	337.1373	1.57	C07655	ESI-	1.8×10^{-7}	1.3×10^{-5}	-18.88	4.8×10^{5}
	Streptamine	C6 H14 N2 O4	178.0948	4.51	C01854	ESI-	4.3×10^{-7}	2.5×10^{-5}	-17.31	1.6×10^{5}
	N,N'-Diacetylchitobiosyldiphosphodolichol	C28 H50 N2 O19 P2	780.2500	4.80	C04537	ESI-	1.8×10^{-7}	1.3×10^{-5}	-16.55	9.6×10^{4}
	UDP-MurNAc(oyl-L-Ala-D-γ-Glu-L-Lys-D-Ala-D-Ala)	C40 H65 N9 O26 P2	1149.3541	11.88	C04702	ESI-	2.6×10^{-5}	7.1×10^{-4}	-16.07	6.9×10^{4}
	Lacto-N-triaose	C20 H35 N O16	545.1931	6.13	—	ESI-	1.1×10^{-5}	3.3×10^{-4}	-15.55	4.8×10^{4}
	Neu5Gc?2-3Gal?1-4GlcNAc?-Sp	C27 H45 N5 O20	759.2639	4.86	—	ESI-	2.3×10^{-3}	4.5×10^{-2}	-1.80	3.5
	D-(+)-Cellobiose	C12 H22 O11	342.1160	4.78	—	ESI-	6.2×10^{-4}	1.4×10^{-2}	-1.73	3.3
**Nucleotides & Nucleosides**	2-Methylguanosine	C11 H15 N5 O5	297.1084	3.02	—	ESI+	2.2×10^{-6}	1.5×10^{-4}	-17.31	1.6×10^{5}
**Vitamins & Cofactors**	MID41949:(6RS)-22-oxo-23,24,25,26,27-pentanorvitamin D3 6,19-sulfur dioxide adduct / (6RS)-22-oxo-23	C22 H32 O4 S	392.2017	1.92	—	ESI-	7.5×10^{-7}	3.7×10^{-5}	-18.16	2.9×10^{5}

Super Class	Compound	Formula	Neutral mass	RT (min)	KEGG ID	ESI	p	p (Corr)	Log FC (45 vs 37)	FC (abs)
	Retinylphosphate mannose	C26 H41 O9 P	528.2502	1.03	—	ESI-	3.1×10^{-7}	2.0×10^{-5}	-16.63	1.0×10^{5}
	dexpanthenol	C9 H19 N O4	205.1314	2.33	C05944	ESI+	1.0×10^{-5}	4.8×10^{-4}	-16.57	9.7×10^{4}
	Precorrin 4	C44 H52 N4 O17	908.3338	4.83	C06407	ESI-	6.4×10^{-8}	7.5×10^{-6}	-16.14	7.2×10^{4}
	Zinc protoporphyrin-9	C34 H32 N4 O4 Zn	624.1701	2.70	C03184	ESI+	2.6×10^{-4}	9.1×10^{-3}	-12.49	5,737.8
	Leucopterin	C6 H5 N5 O3	195.0393	3.06	—	ESI+	5.7×10^{-4}	1.9×10^{-2}	-2.56	5.9
**Fatty Acyls**	13-methyl-hexadecanoic acid	C17 H34 O2	270.2556	0.82	—	ESI-	7.8×10^{-7}	3.8×10^{-5}	-17.98	2.6×10^{5}
	13,14-dihydro-15-keto-tetranor PGE2	C16 H26 O5	298.1776	1.75	—	ESI-	1.2×10^{-4}	3.0×10^{-3}	-17.70	2.1×10^{5}
	epi-4'-hydroxyjasmonic acid	C12 H18 O4	226.1199	1.19	—	ESI+	1.3×10^{-5}	5.8×10^{-4}	-17.59	2.0×10^{5}
	DL-9-hydroxy stearic acid	C18 H36 O3	300.2655	1.17	—	ESI+	3.3×10^{-6}	2.0×10^{-4}	-17.38	1.7×10^{5}
	Dinor-PGD2	C18 H28 O5	324.1951	2.19	—	ESI-	1.7×10^{-5}	4.9×10^{-4}	-17.22	1.5×10^{5}
	Heptadecanoyl Ethanolamide	C19 H39 N O2	313.2978	0.24	—	ESI+	2.2×10^{-5}	8.8×10^{-4}	-16.36	8.4×10^{4}
	8-oxo-nonanoic acid	C9 H16 O3	172.1096	1.04	—	ESI+	1.3×10^{-6}	1.2×10^{-4}	-16.07	6.9×10^{4}
	Lactone of PGF-MUM	C16 H24 O5	296.1609	1.21	—	ESI+	3.2×10^{-7}	8.8×10^{-5}	-15.89	6.1×10^{4}
	7-hydroxy-10-heptadecen-8-ynoic acid	C17 H30 O3	282.2193	1.44	—	ESI+	2.1×10^{-5}	8.6×10^{-4}	-15.80	5.7×10^{4}
	Resolvin D2	C22 H32 O5	376.2241	1.24	C18179	ESI+	9.6×10^{-7}	1.0×10^{-4}	-15.56	4.8×10^{4}
	15-eicosenoic acid	C20 H38 O2	310.2870	1.65	—	ESI-	4.4×10^{-8}	5.8×10^{-6}	-15.23	3.8×10^{4}
	13,14-dihydroxy-docosanoic acid	C22 H44 O4	372.3241	13.44	—	ESI+	1.8×10^{-7}	8.6×10^{-5}	-15.06	3.4×10^{4}
	2R-hydroxy-hexacosanoic acid	C26 H52 O3	412.3901	7.01	—	ESI+	2.5×10^{-7}	8.6×10^{-5}	-13.31	1.0×10^{4}
**Glycerolipids**	Tributyrin	C15 H26 O6	302.1725	3.44	C13870	ESI-	1.9×10^{-5}	5.5×10^{-4}	-16.73	1.1×10^{5}
	DG(18:0/18:0/0:0)	C39 H76 O5	624.5699	1.31	—	ESI+	1.2×10^{-5}	5.4×10^{-4}	-15.35	4.2×10^{4}
	1,2-Dihexanoyl-sn-glycerol	C15 H28 O5	288.1933	6.27	—	ESI-	1.0×10^{-7}	9.1×10^{-6}	-15.08	3.5×10^{4}
**Phospholipids**	1-(8-[3]-ladderane-octanoyl)-2-(8-[3]-ladderane-octanyl)-sn-glycero-3-phospho-(1'-sn-glycerol)	C46 H77 O9 P	804.5291	3.72	—	ESI-	1.4×10^{-4}	3.4×10^{-3}	-19.96	1.0×10^{6}
	PE(O-18:1(9Z)/0:0)	C23 H48 N O6 P	465.3225	2.44	—	ESI+	3.6×10^{-5}	1.4×10^{-3}	-17.78	2.2×10^{5}

Super Class	Compound	Formula	Neutral mass	RT (min)	KEGG ID	ESI	p	p (Corr)	Log FC (45 vs 37)	FC (abs)
	PE(18:0(10(R)Me)/16:0)	C40 H80 N O8 P	733.5634	1.03	—	ESI-	1.9×10^{-7}	1.4×10^{-5}	-16.29	8.0×10^{4}
	CL(1'-[16:0/18:1(11Z)],3'-[16:0/18:1(11Z)])	C77 H146 O17 P2	1405.0021	0.88	—	ESI+	4.0×10^{-6}	2.2×10^{-4}	-15.22	3.8×10^{4}
	PE(22:4(7Z,10Z,13Z,16Z)/19:0)	C46 H84 N O8 P	809.5919	1.04	—	ESI+	3.6×10^{-7}	8.8×10^{-5}	-15.18	3.7×10^{4}
**Sphingolipids**	Glucosylsphingosine	C24 H47 N O7	461.3335	1.38	C03108	ESI-	1.2×10^{-6}	5.4×10^{-5}	-17.67	2.1×10^{5}
	Cer(d18:2/16:0)	C34 H65 N O3	535.4949	1.38	—	ESI+	1.1×10^{-5}	5.2×10^{-4}	-14.46	2.2×10^{4}
**Sterols & Steroids**	17beta-Hydroxyestr-4-en-3-one benzoate	C25 H30 O3	378.2193	4.89	C15171	ESI-	3.5×10^{-8}	5.0×10^{-6}	-16.14	7.2×10^{4}
**Terpenoids**	10-Deacetyl-2-debenzoylbaccatin III	C22 H32 O9	440.2036	1.63	C11899	ESI-	1.9×10^{-7}	1.4×10^{-5}	-18.79	4.5×10^{5}
**Phenolic Compounds**	7,8,4'-Trihydroxy-3',5'-dimethoxyflavanone 4'-O-glucoside	C23 H26 O12	494.1405	6.08	—	ESI-	5.2×10^{-5}	1.3×10^{-3}	-23.78	1.4×10^{7}
	6'''-(3-Hydroxy-3-methylglutaroyl)isoviolanthin	C33 H38 O18	722.2070	4.83	—	ESI-	6.2×10^{-7}	3.2×10^{-5}	-17.53	1.9×10^{5}
	Kolanone	C33 H42 O4	502.3100	1.37	C09948	ESI-	8.4×10^{-8}	8.3×10^{-6}	-16.59	9.9×10^{4}
	Quercetin 3-(2'''-feruloylsophoroside)	C37 H38 O20	802.1981	4.85	—	ESI-	1.7×10^{-7}	1.3×10^{-5}	-16.57	9.7×10^{4}
	Anacardic Acid	C22 H36 O3	348.2667	0.93	C10759	ESI-	1.6×10^{-6}	6.6×10^{-5}	-16.47	9.1×10^{4}
	Acacetin 7-(4''''-Acetylrhamnosyl)-(1-6)-glucosyl-(1-3)-(6''-acetylglucoside)	C38 H46 O21	838.2517	4.33	—	ESI-	1.4×10^{-4}	3.3×10^{-3}	-15.18	3.7×10^{4}
	Altersolanol A	C16 H16 O8	336.0835	3.00	C10296	ESI+	1.9×10^{-6}	1.4×10^{-4}	-15.12	3.6×10^{4}
	Podorhizol beta-D-glucoside	C28 H34 O13	578.2046	2.61	C10876	ESI+	2.5×10^{-7}	8.6×10^{-5}	-13.91	1.5×10^{4}
	4'-O-Methylneobavaisoflavone 7-O-(2''-p-coumaroylglucoside)	C36 H36 O11	644.2208	4.84	—	ESI-	8.2×10^{-5}	2.1×10^{-3}	-2.06	4.2
	Isorhamnetin 3-(6''-malonylglucoside)	C25 H24 O15	564.1124	2.37	—	ESI-	2.3×10^{-3}	4.5×10^{-2}	-1.23	2.3
**Alkaloids**	Longicaudatin	C26 H22 O7	446.1386	3.53	—	ESI-	7.5×10^{-8}	8.1×10^{-6}	-18.24	3.1×10^{5}
	Lophophorine	C13 H17 N O3	235.1213	8.79	C09573	ESI+	1.1×10^{-7}	8.6×10^{-5}	-17.72	2.2×10^{5}
	Akuammine	C22 H26 N2 O4	382.1882	1.52	C09027	ESI+	2.4×10^{-6}	1.6×10^{-4}	-15.74	5.5×10^{4}
**Plant-derived Metabolites**	Glucobrassicin	C16 H20 N2 O9 S2	448.0598	6.65	C05837	ESI+	6.7×10^{-4}	2.2×10^{-2}	-12.11	4,427.3

Super Class	Compound	Formula	Neutral mass	RT (min)	KEGG ID	ESI	p	p (Corr)	Log FC (45 vs 37)	FC (abs)
**Microbiota-derived Metabolites**	N-octanoyl-L-Homoserine lactone	C12 H21 N O3	227.1523	1.26	—	ESI+	6.1×10^{-8}	8.6×10^{-5}	-18.91	4.9×10^{5}

Neutral mass, monoisotopic mass of the neutral molecule; values for features detected as $[M+Na]^+$, $[M+HCOO]^-$ or $[M+CH_3COO]^-$ adducts were corrected to the neutral mass. p, unadjusted t-test p-value; p (Corr), Benjamini–Hochberg adjusted p-value. Log FC, $\log_2$ fold change (45 °C vs 37 °C); FC (abs), absolute fold change.

Very large $|\log_2 FC|$ values arise from features that were detected in one temperature group only and should be read as presence–absence contrasts rather than measured ratios; features detected in all six samples give the smaller, quantitative fold changes.

Annotations are based on accurate mass, isotope pattern and database matching (MSI level 2–3) and were not confirmed with authentic standards. Em dash (—) indicates that the value is not available.
