# Supplementary material for: Metabolomic Profiling Reveals Heat-Responsive Metabolic Markers in Honey Bee Workers Under Contrasting Diets
Source: Int J Mol Sci. 2026 Oct 17;27(20):9237. doi: 10.3390/ijms27209237 (PMC13705331; doi:10.3390/ijms27209237)
Supplement: Supplementary file 1 [file ijms-27-09237-s001.zip › TableS2_S_37vs45.pdf]

Table S2. Complete list of metabolites significantly altered in S workers after heat exposure (45 °C, 3 h) relative to the 37 °C control.

All 123 named metabolites passing |FC| ≥ 2 and Benjamini–Hochberg adjusted $p < 0.05$ in the S (sucrose alone) diet group, ordered by direction of regulation, super class, and $|\log_2 FC|$.

Super Class	Compound	Formula	Neutral mass	RT (min)	KEGG ID	ESI	p	p (Corr)	Log FC (45 vs 37)	FC (abs)
**Up-regulated at 45 °C ↑ (n = 56)**										
**Amino Acids & Peptides**	3-Amino-3-(4-hydroxyphenyl)propanoate	C9 H11 N O3	181.0732	2.88	C04368	ESI-	5.5×10^{-9}	1.4×10^{-5}	22.44	5.7×10^{6}
	Succinylproline	C9 H13 N O5	215.0790	2.07	C11711	ESI-	1.3×10^{-6}	7.0×10^{-5}	19.98	1.0×10^{6}
	N-Acetyl-L-Histidine	C8 H11 N3 O3	197.0791	3.02	C02997	ESI-	1.5×10^{-6}	7.6×10^{-5}	19.06	5.5×10^{5}
	S-Prenyl-L-cysteine	C8 H15 N O2 S	189.0823	2.93	—	ESI-	2.3×10^{-7}	3.4×10^{-5}	18.89	4.9×10^{5}
	Ne-Methyl-L-lysine	C7 H16 N2 O2	160.1211	12.31	C02728	ESI-	1.6×10^{-7}	3.1×10^{-5}	18.29	3.2×10^{5}
	N-Pyruvoyl-5-methoxy-3-hydroxyanthranilate	C11 H11 N O6	253.0590	2.93	C11467	ESI-	3.0×10^{-6}	1.3×10^{-4}	16.77	1.1×10^{5}
	DOPA sulfate	C9 H11 N O7 S	277.0249	1.88	—	ESI-	3.3×10^{-5}	9.2×10^{-4}	15.73	5.4×10^{4}
	Quisqualic acid	C5 H7 N3 O5	189.0392	5.46	C08296	ESI+	1.9×10^{-7}	3.1×10^{-5}	15.33	4.1×10^{4}
	L-Dopa	C9 H11 N O4	197.0685	7.90	C00355	ESI-	1.7×10^{-6}	8.5×10^{-5}	15.03	3.4×10^{4}
	Thiomorpholine 3-carboxylate	C5 H9 N O2 S	147.0356	8.30	C03901	ESI+	3.2×10^{-6}	1.9×10^{-4}	14.91	3.1×10^{4}
	Prephenic acid	C10 H10 O6	226.0482	5.39	C00254	ESI+	6.1×10^{-6}	3.0×10^{-4}	13.73	1.4×10^{4}
**Amines & Polyamines**	n-Pentadecylamine	C15 H33 N	227.2612	0.26	—	ESI+	5.3×10^{-8}	1.5×10^{-5}	17.05	1.4×10^{5}
	N4-Phosphoagmatine	C5 H15 N4 O3 P	210.0882	11.57	C02726	ESI-	8.3×10^{-6}	2.7×10^{-4}	16.56	9.6×10^{4}
	N1,N5,N10-Tricaffeoyl spermidine	C34 H37 N3 O9	631.2561	1.50	C18070	ESI-	3.0×10^{-4}	7.1×10^{-3}	13.10	8,795.3
**Organic Acids**	Mesaconic acid	C5 H6 O4	130.0264	10.95	C01732	ESI-	3.5×10^{-7}	4.1×10^{-5}	20.48	1.5×10^{6}
	Oxolan-3-one	C4 H6 O2	86.0368	10.97	—	ESI-	3.2×10^{-7}	4.1×10^{-5}	19.73	8.7×10^{5}
	Aconitic acid	C6 H6 O6	174.0165	10.98	C00417	ESI-	5.3×10^{-6}	1.8×10^{-4}	18.65	4.1×10^{5}
	2,3-dihydroxy-valeric acid	C5 H10 O4	134.0577	3.10	—	ESI-	8.1×10^{-8}	2.3×10^{-5}	16.93	1.2×10^{5}
**Carbohydrates & Sugars**	D-Mannonate	C6 H12 O7	196.0580	2.51	C00514	ESI-	3.4×10^{-6}	1.3×10^{-4}	21.16	2.3×10^{6}
	1,5-Anhydro-4-deoxy-D-glycero-hex-1-en-3-ulose	C6 H8 O4	144.0423	7.81	C19826	ESI-	2.9×10^{-9}	1.4×10^{-5}	20.74	1.8×10^{6}

Super Class	Compound	Formula	Neutral mass	RT (min)	KEGG ID	ESI	p	p (Corr)	Log FC (45 vs 37)	FC (abs)
	Levan	C18 H32 O16	504.1666	5.59	C01355	ESI-	1.8×10^{-5}	5.2×10^{-4}	15.91	6.2×10^{4}
	2-Deoxy-2,3-dehydro-N-acetylneuraminic acid	C11 H17 N O8	291.0972	7.28	C04580	ESI-	2.5×10^{-6}	1.1×10^{-4}	15.90	6.1×10^{4}
	Lacto-N-tetraose	C26 H45 N O21	707.2484	8.24	C06371	ESI-	1.1×10^{-4}	2.9×10^{-3}	15.56	4.8×10^{4}
	Fucα1-2Galβ1-4GlcNAcβ1-3Galβ1-4GlcNAcβ-Sp	C36 H61 N5 O25	963.3656	11.40	—	ESI-	6.0×10^{-7}	4.7×10^{-5}	15.18	3.7×10^{4}
	Levan (feature 2)	C18 H32 O16	504.1690	2.36	C01355	ESI-	5.8×10^{-4}	1.3×10^{-2}	13.53	1.2×10^{4}
	ADP-Mannose	C16 H25 N5 O15 P2	589.0822	8.51	C06192	ESI-	5.2×10^{-4}	1.2×10^{-2}	13.14	9,004.0
**Nucleotides & Nucleosides**	1-Methyladenine	C6 H7 N5	149.0703	1.53	C02216	ESI+	2.9×10^{-5}	1.1×10^{-3}	20.78	1.8×10^{6}
	1-Methyladenine (feature 2)	C6 H7 N5	149.0700	5.99	C02216	ESI+	1.4×10^{-8}	1.1×10^{-5}	15.78	5.6×10^{4}
**Vitamins & Cofactors**	Axerophthene	C20 H30	270.2349	0.92	—	ESI+	1.9×10^{-8}	1.2×10^{-5}	17.22	1.5×10^{5}
**Fatty Acyls**	11-methyl-tridecanoic acid	C14 H28 O2	228.2089	1.29	—	ESI-	2.2×10^{-7}	3.4×10^{-5}	20.70	1.7×10^{6}
	Δ17-6-keto-PGF1α	C20 H32 O6	368.2198	1.83	—	ESI-	7.2×10^{-7}	5.1×10^{-5}	17.25	1.6×10^{5}
	8,9-DiHETrE-EA	C22 H39 N O4	381.2864	1.07	—	ESI-	4.6×10^{-7}	4.5×10^{-5}	17.07	1.4×10^{5}
	DL-10-hydroxy stearic acid	C18 H36 O3	300.2664	2.61	—	ESI-	9.7×10^{-7}	5.9×10^{-5}	17.05	1.4×10^{5}
	10E,16-heptadecadien-8-ynoic acid	C17 H26 O2	262.1935	1.41	—	ESI+	2.9×10^{-7}	4.1×10^{-5}	17.00	1.3×10^{5}
	Dodecanedioic acid	C12 H22 O4	230.1510	3.01	C02678	ESI-	1.7×10^{-6}	8.7×10^{-5}	16.10	7.0×10^{4}
	Palmitic amide	C16 H33 N O	255.2571	1.76	—	ESI+	1.1×10^{-8}	1.1×10^{-5}	15.93	6.2×10^{4}
	10S,11R-epoxy-punaglandin 4	C25 H35 Cl O9	514.1983	8.62	—	ESI+	1.3×10^{-6}	1.0×10^{-4}	14.83	2.9×10^{4}
**Glycerolipids**	MGDG(18:5(3Z,6Z,9Z,12Z,15Z)/18:4(6Z,9Z,12Z,15Z))	C45 H68 O10	768.4812	0.96	—	ESI-	5.7×10^{-6}	2.0×10^{-4}	14.64	2.6×10^{4}
**Phospholipids**	PI(O-18:0/18:3(6Z,9Z,12Z))	C45 H83 O12 P	846.5643	1.30	—	ESI-	2.8×10^{-7}	3.7×10^{-5}	18.75	4.4×10^{5}
	PE(8:0/8:0)	C21 H42 N O8 P	467.2666	1.44	—	ESI-	1.7×10^{-5}	5.0×10^{-4}	16.50	9.2×10^{4}
**Sphingolipids**	D-erythro-Sphingosine C-15	C15 H31 N O2	257.2348	1.52	—	ESI+	1.9×10^{-6}	1.3×10^{-4}	15.17	3.7×10^{4}
	N-(24-hydroxytetracosanyl)sphinganine	C42 H85 N O4	667.6448	4.58	—	ESI-	2.2×10^{-3}	4.6×10^{-2}	11.99	4,081.4
**Sterols & Steroids**	Evasterioside C	C31 H52 O12 S	648.3179	4.09	—	ESI-	3.5×10^{-7}	4.1×10^{-5}	16.27	7.9×10^{4}

Super Class	Compound	Formula	Neutral mass	RT (min)	KEGG ID	ESI	p	p (Corr)	Log FC (45 vs 37)	FC (abs)
	diginatin	C41 H64 O15	796.4248	2.25	C08861	ESI+	4.5×10^{-6}	2.4×10^{-4}	13.83	1.5×10^{4}
	Estriol 3-sulfate 16-glucuronide	C24 H32 O12 S	544.1621	6.00	—	ESI+	3.1×10^{-6}	1.9×10^{-4}	13.10	8,782.3
**Terpenoids**	(10S)-Juvenile hormone III diol	C16 H28 O4	284.1987	1.39	C16505	ESI-	7.9×10^{-5}	2.1×10^{-3}	22.30	5.2×10^{6}
	capsidiol	C15 H24 O2	236.1772	0.95	C09627	ESI-	7.4×10^{-8}	2.2×10^{-5}	17.84	2.3×10^{5}
**Phenolic Compounds**	6,8-Di-C-beta-D-arabinopyranosylapigenin	C25 H26 O13	534.1385	1.57	—	ESI-	1.4×10^{-8}	1.4×10^{-5}	18.21	3.0×10^{5}
	4-O-Methyl-gallate	C8 H8 O5	184.0378	10.82	—	ESI-	3.0×10^{-7}	3.9×10^{-5}	17.76	2.2×10^{5}
	Epimedokoreanin A	C25 H24 O8	452.1451	4.71	—	ESI+	7.6×10^{-6}	3.6×10^{-4}	15.49	4.6×10^{4}
	Artonol D	C30 H26 O7	498.1679	11.87	—	ESI-	1.4×10^{-7}	3.0×10^{-5}	14.77	2.8×10^{4}
	Nitiducarpin	C26 H26 O5	418.1799	2.26	—	ESI-	1.5×10^{-4}	3.8×10^{-3}	1.58	3.0
**Alkaloids**	Taxine B	C33 H45 N O8	583.3145	1.25	C19989	ESI-	8.6×10^{-6}	2.8×10^{-4}	17.73	2.2×10^{5}
	(S)-Autumnaline	C21 H27 N O5	373.1871	3.03	C16707	ESI-	5.3×10^{-7}	4.7×10^{-5}	16.21	7.6×10^{4}
	Manzamine A	C36 H44 N4 O	548.3546	2.48	C19898	ESI+	1.3×10^{-6}	1.0×10^{-4}	15.76	5.5×10^{4}
	alpha-Ergocryptine	C32 H41 N5 O5	575.2977	4.55	C07545	ESI-	1.4×10^{-4}	3.6×10^{-3}	12.48	5,696.9
**Down-regulated at 45 °C ↓ (n = 67)**										
**Amino Acids & Peptides**	5-Aminopentanoic acid	C5 H11 N O2	117.0790	1.42	C00431	ESI+	7.4×10^{-8}	1.7×10^{-5}	-18.98	5.2×10^{5}
	S-(N-Hydroxy-N-methylcarbamoyl)glutathione	C12 H20 N4 O8 S	380.0993	1.96	C04572	ESI-	2.4×10^{-6}	1.1×10^{-4}	-18.50	3.7×10^{5}
	Ne-Methyl-L-lysine	C7 H16 N2 O2	160.1210	12.04	C02728	ESI-	8.3×10^{-7}	5.4×10^{-5}	-18.24	3.1×10^{5}
	Ac-Tyr-OEt	C13 H17 N O4	251.1162	1.40	C01657	ESI-	4.0×10^{-7}	4.4×10^{-5}	-18.14	2.9×10^{5}
	(E)-2-Butenyl-4-methyl-threonine	C9 H17 N O3	187.1208	1.71	C12029	ESI-	2.2×10^{-7}	3.4×10^{-5}	-17.96	2.5×10^{5}
	Kynurenine	C10 H12 N2 O3	208.0864	2.59	C00328	ESI+	4.7×10^{-5}	1.7×10^{-3}	-17.00	1.3×10^{5}
	(3S)-3,6-Diaminohexanoate	C6 H14 N2 O2	146.1053	11.02	C01142	ESI-	2.2×10^{-7}	3.4×10^{-5}	-16.98	1.3×10^{5}
	L-hydroxy Arginine	C6 H14 N4 O3	190.1060	8.57	—	ESI+	3.0×10^{-8}	1.2×10^{-5}	-15.44	4.4×10^{4}

Super Class	Compound	Formula	Neutral mass	RT (min)	KEGG ID	ESI	p	p (Corr)	Log FC (45 vs 37)	FC (abs)
	Melatonin	C13 H16 N2 O2	232.1201	8.49	C01598	ESI+	5.9×10^{-6}	2.9×10^{-4}	-14.55	2.4×10^{4}
**Amines & Polyamines**	1-Methylhistamine	C6 H11 N3	125.0952	8.32	C05127	ESI+	6.6×10^{-5}	2.3×10^{-3}	-17.16	1.5×10^{5}
	Hordatine A	C28 H38 N8 O4	550.2991	1.42	C08307	ESI-	4.6×10^{-7}	4.5×10^{-5}	-16.19	7.5×10^{4}
	Histamine	C5 H9 N3	111.0795	4.56	C00388	ESI-	4.7×10^{-6}	1.7×10^{-4}	-14.36	2.1×10^{4}
**Organic Acids**	(S)-Acetoin	C4 H8 O2	88.0523	4.29	C01769	ESI-	6.5×10^{-7}	4.9×10^{-5}	-21.62	3.2×10^{6}
	2-Methyleneglutarate	C6 H8 O4	144.0421	7.80	C02930	ESI-	1.8×10^{-7}	3.2×10^{-5}	-19.28	6.4×10^{5}
	Mesaconic acid	C5 H6 O4	130.0267	11.12	C01732	ESI-	2.6×10^{-6}	1.1×10^{-4}	-18.31	3.2×10^{5}
	Oxolan-3-one	C4 H6 O2	86.0368	11.17	—	ESI-	1.5×10^{-5}	4.6×10^{-4}	-17.38	1.7×10^{5}
**Carbohydrates & Sugars**	4-(4-Deoxy-β-D-gluc-4-enuronosyl)-D-galacturonate	C12 H16 O12	352.0639	1.62	C06118	ESI-	9.8×10^{-8}	2.6×10^{-5}	-20.95	2.0×10^{6}
	1,4-beta-D-Glucan	C18 H32 O18	536.1591	3.99	C00760	ESI-	8.9×10^{-6}	2.9×10^{-4}	-20.87	1.9×10^{6}
	L-Galactono-1,4-lactone	C6 H10 O6	178.0480	1.89	C01115	ESI-	2.6×10^{-7}	3.7×10^{-5}	-18.09	2.8×10^{5}
	Acetyl-maltose	C14 H24 O12	384.1261	6.52	C02130	ESI-	1.8×10^{-5}	5.2×10^{-4}	-16.07	6.9×10^{4}
**Nucleotides & Nucleosides**	dIMP	C10 H13 N4 O7 P	332.0534	1.56	C06196	ESI-	1.4×10^{-4}	3.4×10^{-3}	-20.65	1.6×10^{6}
**Vitamins & Cofactors**	Bilirubin glucuronide	C39 H44 N4 O12	760.2974	1.44	C03033	ESI-	1.4×10^{-3}	3.1×10^{-2}	-14.08	1.7×10^{4}
**Fatty Acyls**	13,14-Dihydro-15-keto-tetranor-PGF1α	C16 H28 O5	300.1931	2.55	—	ESI-	1.7×10^{-5}	5.0×10^{-4}	-19.08	5.5×10^{5}
	4Z-decenoic acid	C10 H18 O2	170.1308	1.20	—	ESI+	1.7×10^{-8}	1.1×10^{-5}	-17.51	1.9×10^{5}
	Δ17-6-keto-PGF1α	C20 H32 O6	368.2200	1.33	—	ESI-	7.4×10^{-6}	2.5×10^{-4}	-17.23	1.5×10^{5}
	Tricosanyl oleate	C39 H76 O2	576.5823	0.99	—	ESI-	7.3×10^{-7}	5.1×10^{-5}	-17.05	1.4×10^{5}
	10-hydroxy-hexadecan-1,16-dioic acid	C16 H30 O5	302.2089	2.09	—	ESI-	7.3×10^{-6}	2.4×10^{-4}	-16.99	1.3×10^{5}
	1-tetradecanol	C14 H30 O	214.2299	1.02	—	ESI+	1.2×10^{-8}	1.1×10^{-5}	-16.94	1.3×10^{5}
	13,14-dihydro-19(R)-hydroxyPGE1	C20 H36 O6	372.2498	1.94	—	ESI-	1.3×10^{-6}	7.0×10^{-5}	-16.11	7.1×10^{4}
	3R-Methylheptanoic acid	C8 H16 O2	144.1152	4.06	—	ESI-	6.0×10^{-6}	2.1×10^{-4}	-15.58	4.9×10^{4}

Super Class	Compound	Formula	Neutral mass	RT (min)	KEGG ID	ESI	p	p (Corr)	Log FC (45 vs 37)	FC (abs)
	(7Z,10Z,13Z,16Z,19Z)-Docosapentaenoyl-CoA	C43 H68 N7 O17 P3 S	1079.3605	5.09	C16166	ESI-	4.2×10^{-7}	4.4×10^{-5}	-14.84	2.9×10^{4}
**Phospholipids**	PE(19:0/0:0)	C24 H50 N O7 P	495.3331	1.30	—	ESI+	1.4×10^{-5}	6.0×10^{-4}	-18.22	3.1×10^{5}
	PI(20:1(11Z)/14:0)	C43 H81 O13 P	836.5395	1.03	—	ESI-	4.1×10^{-4}	9.3×10^{-3}	-17.25	1.6×10^{5}
	PI(18:4(6Z,9Z,12Z,15Z)/14:0)	C41 H71 O13 P	802.4654	2.02	—	ESI+	4.6×10^{-5}	1.7×10^{-3}	-16.49	9.2×10^{4}
	PE(17:1(9Z)/0:0)	C22 H44 N O7 P	465.2811	2.22	—	ESI+	2.0×10^{-6}	1.4×10^{-4}	-16.21	7.6×10^{4}
	PS(22:4(7Z,10Z,13Z,16Z)/0:0)	C28 H48 N O9 P	573.3034	6.69	—	ESI-	7.4×10^{-5}	1.9×10^{-3}	-15.86	6.0×10^{4}
	Phenolic phosphate	C6 H7 O4 P	174.0081	0.80	C02734	ESI-	7.6×10^{-5}	2.0×10^{-3}	-15.69	5.3×10^{4}
	PA(15:1(9Z)/0:0)	C18 H35 O7 P	394.2120	1.66	—	ESI+	1.8×10^{-6}	1.3×10^{-4}	-15.65	5.2×10^{4}
	PI(16:0/14:0)	C39 H75 O13 P	782.4945	5.85	—	ESI-	2.2×10^{-4}	5.4×10^{-3}	-15.61	5.0×10^{4}
	PS(22:6(4Z,7Z,10Z,13Z,16Z,19Z)/18:4(6Z,9Z,12Z,15Z))	C46 H70 N O10 P	827.4778	2.10	—	ESI+	8.0×10^{-7}	7.2×10^{-5}	-15.08	3.5×10^{4}
	PI(17:1(9Z)/17:1(9Z))	C43 H79 O13 P	834.5254	1.05	—	ESI-	3.3×10^{-6}	1.3×10^{-4}	-14.80	2.9×10^{4}
	PS(15:0/18:2(9Z,12Z))	C39 H72 N O10 P	745.4894	1.86	—	ESI-	4.5×10^{-6}	1.7×10^{-4}	-14.79	2.8×10^{4}
	PI(O-16:0/13:0)	C38 H75 O12 P	754.4901	1.91	—	ESI+	3.0×10^{-6}	1.9×10^{-4}	-14.39	2.1×10^{4}
	PI(18:3(9Z,12Z,15Z)/0:0)	C27 H47 O12 P	594.2807	4.42	—	ESI+	7.5×10^{-4}	2.4×10^{-2}	-1.55	2.9
**Sphingolipids**	Dehydrophytosphingosine	C18 H37 N O3	315.2778	1.78	—	ESI+	9.6×10^{-7}	8.1×10^{-5}	-22.27	5.1×10^{6}
	Myriocin	C21 H39 N O6	401.2761	3.00	C19914	ESI-	1.2×10^{-5}	3.7×10^{-4}	-14.54	2.4×10^{4}
**Sterols & Steroids**	6α-Glucuronosylhyodeoxycholate	C30 H48 O10	568.3239	3.38	C11246	ESI-	6.0×10^{-6}	2.1×10^{-4}	-17.11	1.4×10^{5}
	3α-Hydroxy-12-oxo-5β-chol-8-en-24-oic acid	C24 H36 O4	388.2607	1.57	—	ESI+	8.6×10^{-7}	7.6×10^{-5}	-16.22	7.6×10^{4}
	Proscillaridin A	C30 H42 O8	530.2866	2.03	C12816	ESI-	2.5×10^{-6}	1.1×10^{-4}	-15.32	4.1×10^{4}
	Δ8,14-Sterol	C29 H46 O	410.3536	3.95	C11508	ESI+	1.7×10^{-6}	1.3×10^{-4}	-14.78	2.8×10^{4}
	Urocortisone	C21 H32 O5	364.2215	3.88	C05470	ESI+	4.7×10^{-6}	2.5×10^{-4}	-13.92	1.5×10^{4}
**Terpenoids**	5-(2-hydroxyethyl)isolongifol-4-ene	C17 H28 O	248.2133	1.22	—	ESI+	1.1×10^{-6}	8.9×10^{-5}	-17.60	2.0×10^{5}

Super Class	Compound	Formula	Neutral mass	RT (min)	KEGG ID	ESI	p	p (Corr)	Log FC (45 vs 37)	FC (abs)
	Inucrithmin	C17 H14 O8	346.0674	1.67	—	ESI-	6.8×10^{-7}	5.0×10^{-5}	-17.26	1.6×10^{5}
	Artabsin	C15 H20 O3	248.1413	1.62	C09301	ESI-	3.6×10^{-7}	4.2×10^{-5}	-15.68	5.2×10^{4}
	Trichilin A	C35 H46 O13	674.2956	1.75	C08786	ESI-	1.1×10^{-4}	2.9×10^{-3}	-15.58	4.9×10^{4}
	Fusicoccin A	C36 H58 O12	682.3920	2.14	—	ESI-	8.4×10^{-5}	2.2×10^{-3}	-15.16	3.6×10^{4}
**Phenolic Compounds**	Orsellinic acid	C8 H8 O4	168.0415	2.51	C01839	ESI-	1.1×10^{-6}	6.6×10^{-5}	-18.69	4.2×10^{5}
	Carpachromene dimethyl ether	C22 H20 O5	364.1296	1.51	—	ESI-	1.0×10^{-6}	6.1×10^{-5}	-17.74	2.2×10^{5}
	Methylorsellinic Acid, Ethyl Ester	C11 H14 O4	210.0882	11.32	—	ESI-	1.3×10^{-5}	4.0×10^{-4}	-15.85	5.9×10^{4}
	Quercetin 3-(2''-sinapoylglucoside)-3'-(6''-sinapoylglucoside)-4'-glucoside	C55 H60 O30	1200.3104	7.47	—	ESI-	3.2×10^{-6}	1.3×10^{-4}	-15.55	4.8×10^{4}
	Chalconaringenin 2'-O-glucoside 4'-O-gentobioside	C33 H42 O20	758.2297	2.03	—	ESI-	3.1×10^{-6}	1.3×10^{-4}	-15.24	3.9×10^{4}
	Ammothamnidin	C25 H28 O5	408.1921	2.23	—	ESI+	6.1×10^{-5}	2.2×10^{-3}	-15.02	3.3×10^{4}
	Okanin 4'-O-(2''-O-caffeoyl-6''-O-p-coumaroylglucoside)	C39 H34 O16	758.1849	4.39	—	ESI-	5.5×10^{-7}	4.7×10^{-5}	-14.61	2.5×10^{4}
	Cassiaside B2	C39 H52 O25	920.2821	11.85	C17674	ESI-	2.1×10^{-6}	1.0×10^{-4}	-13.59	1.2×10^{4}
**Alkaloids**	Reserpine	C33 H40 N2 O9	608.2725	3.99	C06539	ESI-	3.7×10^{-7}	4.3×10^{-5}	-18.55	3.8×10^{5}
	Ryanodine	C25 H35 N O9	493.2299	4.92	C08705	ESI-	4.0×10^{-5}	1.1×10^{-3}	-16.78	1.1×10^{5}
	2,6-Dihydroxypseudooxynicotine	C10 H14 N2 O3	210.1001	8.31	C15986	ESI-	1.8×10^{-7}	3.2×10^{-5}	-15.56	4.8×10^{4}

Neutral mass, monoisotopic mass of the neutral molecule; values for features detected as $[M+Na]^+$, $[M+HCOO]^-$ or $[M+CH_3COO]^-$ adducts were corrected to the neutral mass. p, unadjusted t-test p-value; p (Corr), Benjamini–Hochberg adjusted p-value. Log FC, $\log_2$ fold change (45 °C vs 37 °C); FC (abs), absolute fold change.

Very large $|\log_2 FC|$ values arise from features that were detected in one temperature group only and should be read as presence–absence contrasts rather than measured ratios; features detected in all six samples give the smaller, quantitative fold changes.

Annotations are based on accurate mass, isotope pattern and database matching (MSI level 2–3) and were not confirmed with authentic standards. Em dash (—) indicates that the value is not available.
